# Recent Development in Topological Polymer Electrolytes for Rechargeable Lithium Batteries

**DOI:** 10.1002/advs.202206978

**Published:** 2023-03-31

**Authors:** Yu Liu, Qinghui Zeng, Zhenfeng Li, Anqi Chen, Jiazhu Guan, Honghao Wang, Shi Wang, Liaoyun Zhang

**Affiliations:** ^1^ School of Chemical Sciences University of Chinese Academy of Sciences Beijing 100049 China; ^2^ State Key Laboratory of Organic Electronics & Information Displays (SKLOEID) and Institute of Advanced Materials (IAM) Nanjing University of Posts and Telecommunications 9 Wenyuan Road Nanjing 210023 China

**Keywords:** development, lithium‐ion batteries, solid polymer electrolytes, topological polymer matrices

## Abstract

Solid polymer electrolytes (SPEs) are still being considered as a candidate to replace liquid electrolytes for high‐safety and flexible lithium batteries due to their superiorities including light‐weight, good flexibility, and shape versatility. However, inefficient ion transportation of linear polymer electrolytes is still the biggest challenge. To improve ion transport capacity, developing novel polymer electrolytes are supposed to be an effective strategy. Nonlinear topological structures such as hyperbranched, star‐shaped, comb‐like, and brush‐like types have highly branched features. Compared with linear polymer electrolytes, topological polymer electrolytes possess more functional groups, lower crystallization, glass transition temperature, and better solubility. Especially, a large number of functional groups are beneficial to dissociation of lithium salt for improving the ion conductivity. Furthermore, topological polymers have strong design ability to meet the requirements of comprehensive performances of SPEs. In this review, the recent development in topological polymer electrolytes is summarized and their design thought is analyzed. Outlooks are also provided for the development of future SPEs. It is expected that this review can raise a strong interest in the structural design of advanced polymer electrolyte, which can give inspirations for future research on novel SPEs and promote the development of next‐generation high‐safety flexible energy storage devices.

## Graphical abstract

The high designability, flexibility, and abundant functional groups of topological polymer electrolyte matrix are beneficial to enhancing ion conduction and battery performance. This review introduces development of different types of topological solid polymer electrolytes (SPEs), including hyperbranched, star‐shaped, comb‐like, and brush‐like SPEs. Besides, the review analyzes design thoughts of SPEs for promoting the progress of high‐safety flexible energy storage devices.

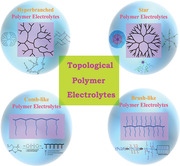

## 1 Introduction

**Figure 1:**
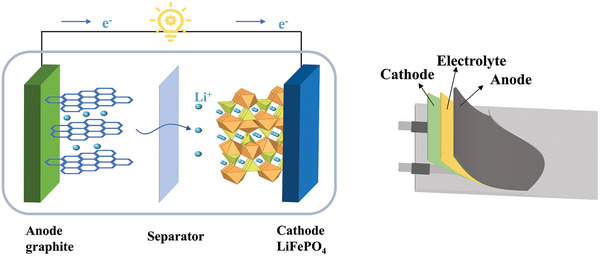
The structures of liquid lithium‐ion battery and flexible lithium‐ion battery.

Energy crisis not only restricts social and economic development but also influences the ecological environment with massive consumption of fossil fuels. To reduce CO_2_ emission from burning fossil fuels, specific attention is paid to the sustainable energy storage devices which are low‐cost, environmentally friendly, and with high energy density.^[^ ^1^, ^2^ ^]^ Among all current energy storage devices, lithium‐ion batteries (LIBs) have received tremendous attention because of their many advantages such as lightweight, long service life, and minimal memory effects since it was first commercialized in the early 1990s by the Sony Corporation.^[^ ^3^, ^4^, ^5^, ^6^ ^]^ Since then, LIBs in portable consumer electronics and electric vehicles have made continuous development and have a great impact on our daily life.^[^ ^7^, ^8^, ^9^, ^10^ ^]^ As for LIBs, a complete commercial liquid lithium‐ion battery system is significantly composed of the cathode, anode, liquid electrolyte, and separator (**Figure** **1**). Being an essential part of LIBs, the electrolyte plays the role of transporting ions between the cathode and anode, which is crucial to influence the performance of the entire battery. Currently, electrolyte is dominantly liquid electrolyte composed of organic solvent and lithium salt. Unfortunately, the liquid electrolytes which have high volatility, flammability, and potential leakage can trigger a series of safety issues, including short‐circuit, overcharging, and thermal runaway.^[^ ^11^, ^12^ ^]^ These possible risks can reduce the performance and service life of the battery to some extent. In addition, constrained by the inherent volume of the liquid electrolyte, traditional liquid LIBs cannot satisfy the needs of microminiaturization and flexibility of next‐generation high‐energy‐density electronic devices. Therefore, it is vital to find reliable material to replace traditional liquid electrolytes to address these issues.

In this regard, the replacement of organic liquid electrolytes with safer solid‐state electrolytes is an effective approach to enhance the performance of batteries. Among many types of solid‐state electrolytes, solid polymer electrolytes (SPEs) have received widespread attention due to their outstanding properties such as good flexibility, lightweight, easy design, and fabrication.^[^ ^13^, ^14^, ^15^ ^]^ Up to now, linear polymer electrolytes complexed or dissolved with alkali metal salt have been thoroughly studied.^[^ ^3^ ^]^ Polyethylene oxide (PEO), the first found ion‐conducting polymer, was discovered by Wright in 1973.^[^ ^16^ ^]^ Then, Armand reported the practical application of the first all‐solid‐state battery equipped with PEO‐based polymer electrolyte membrane in 1979. Hereafter, different linear polymer electrolyte matrices have been developed successively such as polycarbonates, polyurethane, polyacrylate, fluoropolymer, and polyesters. Nevertheless, the bottleneck of these linear polymer electrolytes with low ionic conductivity remains, hindering their further commercialization in future lithium battery applications. Specifically, the extensively studied PEO‐based linear polymer electrolytes are fully affected by inherent crystallization and poor mechanical properties, which manifests the low ionic conductivity at ambient temperature (10^−7^–10^−6^ S cm^−1^) and poor mechanical properties of electrolyte membranes in the melted state.^[^ ^17^, ^18^, ^19^, ^20^, ^21^, ^22^ ^]^ Therefore, it is difficult for these linear polymer electrolytes to achieve the goal of large‐scale commercialization.

For practical purpose, SPEs should satisfy some specific requirements as follows: 1) high ionic conductivity at ambient temperature (>10^−4^ S cm^−1^).^[^ ^17^ ^]^ As an electrolyte, it must have high ionic conductivity and electronic insulation so that it can function as an ion transmission medium while reducing its self‐discharge; 2) good mechanical properties.^[^ ^23^, ^24^ ^]^ Owing to the direct contact with the cathode and anode, the SPEs should possess high mechanical strength to withstand the stress variation from the external environment, while inhibiting the lithium dendrite growth and puncture; 3) applicable Li‐ion transference numbers.^[^ ^25^ ^]^ A high lithium ion transference number can reduce the concentration polarization during the cell cycling, realizing a high energy and power density; 4) wider electrochemical window.^[^ ^26^ ^]^ The electrolyte with wide electrochemical window can be adapted to high voltage electrodes for potential applications; 5) Chemical and thermal stability.^[^ ^26^ ^]^ Good stability can further avoid the possibility of side reactions and thermal decomposition under normal work of the batteries. However, it is arduous to prepare such a linear polymer electrolyte having all of the above properties simultaneously.

Generally speaking, the ionic conductivity of polymer electrolyte is a key parameter to determine the application of polymer electrolyte. How to improve the ionic conductivity has become a great challenge for polymer electrolyte. As for ionic conductivities, the relationship between ionic conductivities and its influence factors can be well described by the Nernst‐Einstein Equation. According to the theoretical formula:

(1) $$\sigma =D\times \frac{Nq^{2}}{kT}$$

where *N* is ionic concentration, *q* is the ionic charge, *k* is Boltzmann constant, and *D* is ion diffusion coefficient, respectively. It is obvious that promoting ionic concentration is an important method to enhance ionic conductivity of the polymer electrolyte.

To realize the promotion of ionic concentration, an effective approach is to change the original linear structure of polymers into the topology branching structure for transporting more lithium‐ions in the same conditions.^[^ ^27^, ^28^ ^]^ In this regard, nonlinear topological polymers are appealing alternative SPE matrices of linear polymers owing to its incomparable advantages. The nonlinear topological structure is rich in functional groups, which can provide more binding sites to dissolve lithium salts. Besides, the nonlinear topological structure is not easy to crystallization, which is more conducive to the conduction of lithium ions. Moreover, nonlinear topological polymers can have more highly flexible segments. The freer movement of polymer segments is capable of enhancing ionic conductivity. In another aspect, the design capacity of nonlinear topological polymer is strong. The introduction of rigid segments into the structure of nonlinear topological polymer is beneficial for improving the mechanical properties of polymer electrolytes. Hence, polymer electrolytes with nonlinear topological architecture have exhibited remarkable characteristics for the improvement of SPEs.

**Figure 2:**
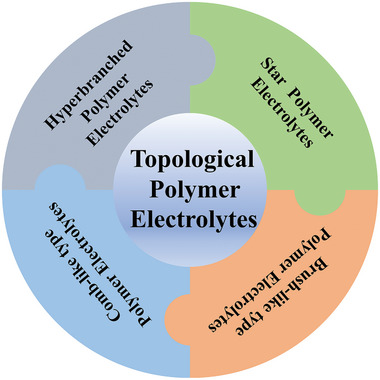
The types of introduced topological polymer electrolytes.

In this article, the recent development in hyperbranched/star‐shaped and comb/brush‐like type polymer electrolytes for rechargeable lithium batteries is reviewed in detail. Here, different types of topological polymer electrolytes (**Figure** 2) are introduced for highlighting the unique contribution of topological structures to improve the comprehensive performance of SPEs. At last, outlooks are also provided for the future development of SPEs. Solid‐state polymer electrolytes are an important direction for future electrolyte development due to their high safety, flexibility, and designability. The nonlinear topological polymer electrolyte is an integral part of solid‐state polymer electrolytes, and it has great potential for developing flexible wearable batteries and more practical applications.

## 2 Hyperbranched Polymer Electrolytes

It is well known that linear polymers such as PEO and polypropylene oxide are the earliest used polymer matrices of SPEs. Due to the high crystallinity of linear polymers, it is difficult to get high ionic conductivity for practical applications. To resolve this, topological systems have been developed in recent decades and have demonstrated its ability to enhance the electrochemical properties of polymer electrolytes. Its special topology architecture restricts the crystallization of polymers and is beneficial to the migration of charge carriers. Hyperbranched polymers are the most typical representatives of topological polymers. They are a class of highly branched polymers with a 3D spherical structure in which history can be traced back to the late 19th century. Furthermore, its molecules show very different properties from the corresponding linear one, such as low viscosity, good solubility, and having a large number of modifiable functional groups. Also, the presence of numerous branching points in the molecule can inhibit the regular arrangement of the polymer chain and make it difficult to crystallize, thereby improving the conductivity of the polymer electrolyte. Hence, hyperbranched polymers are considered to be one of the most promising polymer electrolyte matrices and have been extensively studied.

Recently, various approaches have been proposed to develop numerous types of hyperbranched polymer electrolytes with different structural characteristics, including polyether, polyester, polyurethane, etc.^[^ ^29^, ^30^, ^31^ ^]^ Here, the design thought and advantages of the single‐component hyperbranched polymer electrolytes and the composite one are discussed in this section.

### 2.1 Single‐Component Hyperbranched Polymer Electrolytes

This type of hyperbranched polymer electrolytes contains plenty of polar atoms or groups to facilitate the dissolution, dissociation, and conduction of the lithium salt and is without any other filler in the polymer matrix. Distinguished by different polymer backbones, single‐component hyperbranched polymer electrolytes can be divided into the following categories.

#### 2.1.1 Polyether Backbone

**Figure 3:**
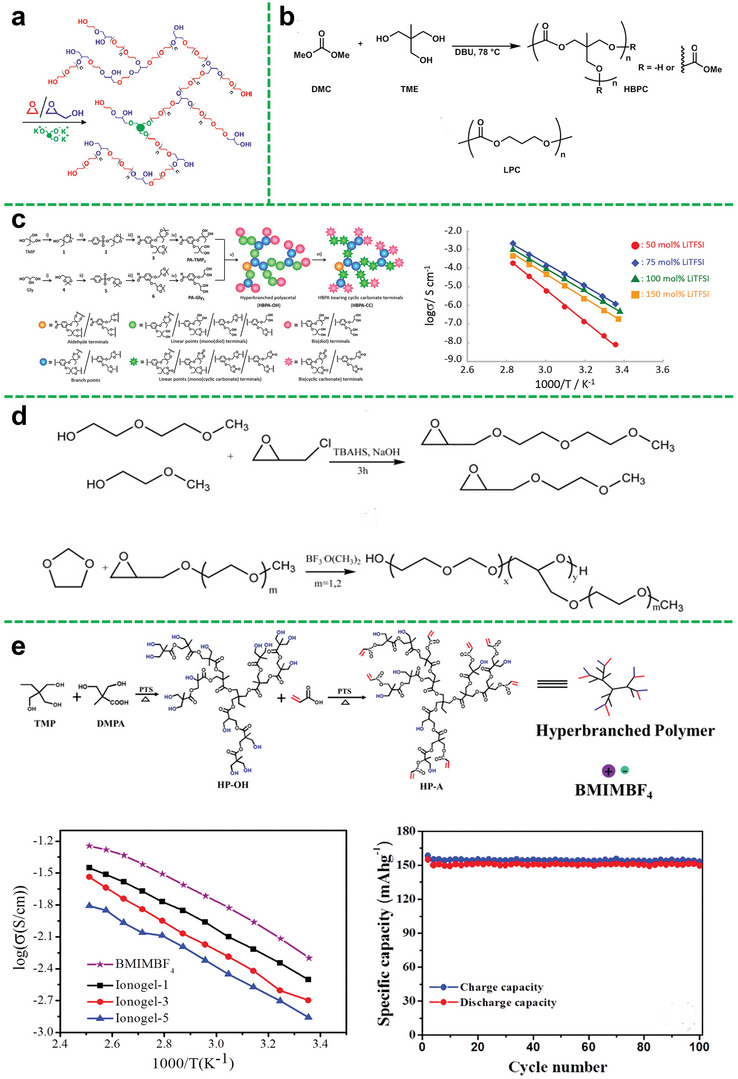
a) Schematic diagram of the synthesis of hyperbranched PEO with varying glycerol units. Reproduced with permission.^[^ ^32^ ^]^ Copyright 2011, American Chemical Society. b) Synthesis of a hyper‐branched polycarbonate (HBPC) from dimethyl carbonate (DMC) and trimethylolethane (TME). Reproduced with permission.^[^ ^34^ ^]^ Copyright 2018, Elsevier Ltd. c) Synthetic route to HBPAs bearing diol terminals (HBPA‐OHs) and HBPAs having CC terminals (HBPA‐CCs) from TMP and Gly under phosgene‐free conditions; temperature dependences of ion conductivity of HBPA‐CC‐4/LiTFSI hybrid films prepared at different CC/LiTFSI ratios. Reprinted with permission.^[^ ^35^ ^]^ Copyright 2019, Wiley Periodicals, Inc. d) Synthetic route of the copolymer. Reproduced with permission.^[^ ^43^ ^]^ Copyright 2014, Elsevier B.V. All rights reserved. e) Schematic illustration of the synthesis of hyperbranched aliphatic polyester (HP‐A); temperature dependence of ionic conductivities for neat BMIMBF_4_ and ionogel shown in the Arrhenius convention; cycling performance of the Li/ionogels/LiFePO_4_ type cell with a charge‐discharge rate of 0.1 C at room temperature. Reproduced with permission.^[^ ^30^ ^]^ Copyright 2019, Elsevier Ltd.

Polyethers like PEO are the earliest and most widely used polymer electrolyte matrix in previous studies, which has a good affinity to lithium salts and exhibits good compatibility toward lithium metal. Also, a large number of the ether‐oxygen segments of hyperbranched polyether promote the ion conduction. In this regard, Lee et al. studied the relationship of the ionic conductivity in a hyperbranched structure and the branching degree as well as the end group functionality.^[^ ^32^ ^]^ They demonstrated that the unique hyperbranched poly (ethylene oxide) polymer (hbPEO) electrolytes with the ability to prevent crystallization showed an increased ionic conductivity of 6 × 10^−5^ S cm^−1^ at ambient conditions (**Figure** **3**a). This novel designing strategy using hyperbranched structure for the enhancement of ionic mobility sheds light on the preparation of SPEs with improved electrochemical properties.

Besides, there are many factors would influence the electrochemical performance, like the molecular weight of polymer and degree of branch. Generally, the higher the molecular weight and the degree of branching, the polymer electrolytes will have more functional groups in dissolving lithium salts, which is beneficial to improving ion conductivity. Tobias Abrahamsson et al. has been studied this in their work.^[^ ^33^ ^]^ They controlled the polymer size by selecting materials with different molecular weights. According to the results, the ionic conductivity has been improved by increasing the polymer size. In addition, when the molecular weight of topological polymer electrolyte matrix is high, the synthesized electrolyte usually has higher mechanical strength. The high mechanical strength of the electrolyte is beneficial to suppressing the formation of lithium dendrites during cell cycling, which is also essential to ensure a battery with sufficient security. Besides, low molecular weight of topological polymer is mostly too viscous to prepare the self‐standing electrolyte membranes, increasing the molecular weight of polymer electrolyte matrix is conducive to improving film‐forming property. Therefore, the choice of molecular weight of polymer is also very important for the property of polymer electrolyte and even for the performance of battery.

#### 2.1.2 Polycarbonate Backbone

To obtain high‐performance polymer electrolytes at specific temperature range, elaborate designing and effective selection of polymer functional groups and segment structure are required to effectively weaken the anion‐cation interaction. Hence, amorphous polymers with good segment flexibility are an ideal class of polymer electrolyte matrix materials, and aliphatic polycarbonate is one of them. In particular, polycarbonate with strongly polar carbonate groups can also be used to synthesize hyperbranched polymer electrolytes in addition to polyethers. Compared with hyperbranched polyether electrolyte, hyperbranched polycarbonate electrolyte not only facilitates the dissociation of lithium salt by interaction between a large of polar carbonate groups with lithium ion of salt, but weaker interaction between the carbonate group and lithium ions is more beneficial to effectively increasing the number of free lithium ions and improving ion conductivity. Suguru et al. reported an aliphatic hyper‐branched polycarbonate (HBPC) (Figure 3b) as a novel host material for SPEs through the polycondensation.^[^ ^34^ ^]^ This hyper‐branched polycarbonate structure was also beneficial for improving the ion transmission and achieving lower activation energy of 11.47 kJ mol^−1^. Specifically, the ionic conductivity of the HBPC‐based SPE reached its maximum values of 1.86 × 10^−4^ S cm^−1^ at 70 °C and 8.52 × 10^−6^ S cm^−1^ at 30 °C. Besides, the HBPC‐based SPE with the preponderant structure exhibited remarkable ionic conductivity as compared with the linear one in a wider temperature range. Therefore, it also indicates that the introduction of hyper‐branched structure in polycarbonates is beneficial for promoting lithium ion transport.

#### 2.1.3 Polyacetal Backbone

Polyacetal has also been reported as a material for preparing hyperbranched polymer electrolytes, which is obtained by the reaction of aldehyde/ketone and alcohol. For hyper‐branched polymer electrolytes, it is actual the end group that plays a decisive role in promoting the ionic conductivity. As shown in Figure 3c, Matsukizono et al. designed a hyperbranched polyacetals (HBPAs) bearing cyclic carbonate (CC) terminals that were synthesized from protocatechuric aldehydes bearing bifunctional trimethylolpropane (TMP) or glycerol (Gly) structures.^[^ ^35^ ^]^ In this study, the end group is the aldehyde, which is beneficial for ion transport, and the authors utilized these HBPA‐CCs with LiTFSI to form uniform electrolyte films. The ion conductivity of the membrane could achieve 2.1 × 10^−3^ S cm^−1^ at 80 °C.

Although the single‐component hyperbranched polymer electrolyte can inhibit polymer crystallization and provide more functional groups, which is good for improving ionic conductivity, good comprehensive performances of the single‐component hyperbranched polymer electrolyte required for the practical application still need to be strengthened. It is an effective approach to introducing other functional component into the hyperbranched systems as the additives to further enhance the overall performance.

### 2.2 Composite Hyperbranched Polymer Electrolytes

The composite method is a film‐fabrication process in which multi‐component ingredients are compounded together to obtain a homogeneous composite electrolyte system, combining the excellent characteristics of different components. In this regard, compounding hyperbranched polymers with a different polymer matrix or modifying the commercialized polyolefin separators is the representative example.

#### 2.2.1 Modification of Polyolefin Separators

The existing polyolefin separators have excellent mechanical strength and chemical stability, but the poor wettability with liquid electrolytes limits its further applications. It was reported that an effective physical modification method for improving the overall properties of polyolefin separators is to incorporate polymers.^[^ ^36^, ^37^, ^38^, ^39^, ^40^, ^41^ ^]^ Xue et al. indicated that the combination of hyperbranched polybenzimidazole (HBPBI) with commercial polyethylene (PE) separator could exhibit superior performance because of the enhanced interface compatibility and ion conductivity.^[^ ^42^ ^]^ Consequently, the wettability, electrolyte uptake, heat resistance, and thermal stability of the obtained separator have been greatly improved by adding the reactive functional groups such as benzimidazole, amino, and carboxyl groups. The cross‐linked hyperbranched structure bearing groups with excellent wettability was introduced into porous PE separator, showing much‐improved diffusion mobility of Li‐ions. For instance, the ionic conductivity of the modified separator was 0.77 mS cm^−1^ at 25 °C, which was almost 10.6 times the value of a commercial PE separator as a control. Furthermore, the introduction of the modified layer onto the surface of PE separator resulted in excellent cell performance, in which discharge capacities were 140 mAh g^−1^ (the control cells were 133 mAh g^−1^) at 0.1 C rate. Besides, the discharge capacity retention rate was 91.1% after 400 cycles at 2 C rate and 73% increase in power capability at 7 C rate. Thus, the effective combination of porous polyolefin separators and multifunctional hyperbranched polymers could realize the improvement of overall comprehensive performance.

#### 2.2.2 Polymer‐Polymer

For the modification of polyolefin separators, the separators mainly support the polymer, but for ion conduction, the contribution is limited. And the polyolefin separators and some polymers do not have a great compatibility. So that we can choose another polymer that has great strength for supporting to replace the polyolefin separators, which can improve not only the ion transport capacity, but also the compatibility. Zheng et al. chose the branched copolyethers (Figure 3d) as the additives to prepare the composite polymer electrolyte.^[^ ^43^ ^]^ Benefiting from the good compatibility between these copolymers and the PEO matrix as well as the negligible crystallization, the ion conductivity could be greatly improved. Thus, the maximum value of ionic conductivity of the composite polymer electrolyte was 2.2 × 10^−4^ S cm^−1^ at 30 °C.

Likewise, a new family of chemical cross‐linked hyperbranched gel electrolytes was successfully synthesized by hyperbranched aliphatic (HP‐A) polyester (Figure 3e), Polyvinylidene fluoride (PVDF), and ionic liquid of 1‐butyl‐3‐methylimidazolium tetrafluoroborate (BMIMBF_4_).^[^ ^30^ ^]^ Importantly, the branching structure of hyperbranched aliphatic polyester can possess higher segmental motion ability and enhanced mechanical properties, resulting in relatively high ionic conductivity and moderate mechanical strength. Thus, the prepared gel electrolyte exhibited high mechanical strength of 1.6 MPa and high mechanical stability even at temperatures up to 200 °C. The superior ionic conductivity showed a wide temperature range from 1.2 × 10^−3^ S cm^−1^ at 20 °C up to 5.0 × 10^−2^ S cm^−1^ at 120 °C. Moreover, the assembled battery displayed a higher specific capacity of 153.1 mAh g^−1^ and retained 98.1% after 100 cycles, as shown in Figure 3e.

**Figure 4:**
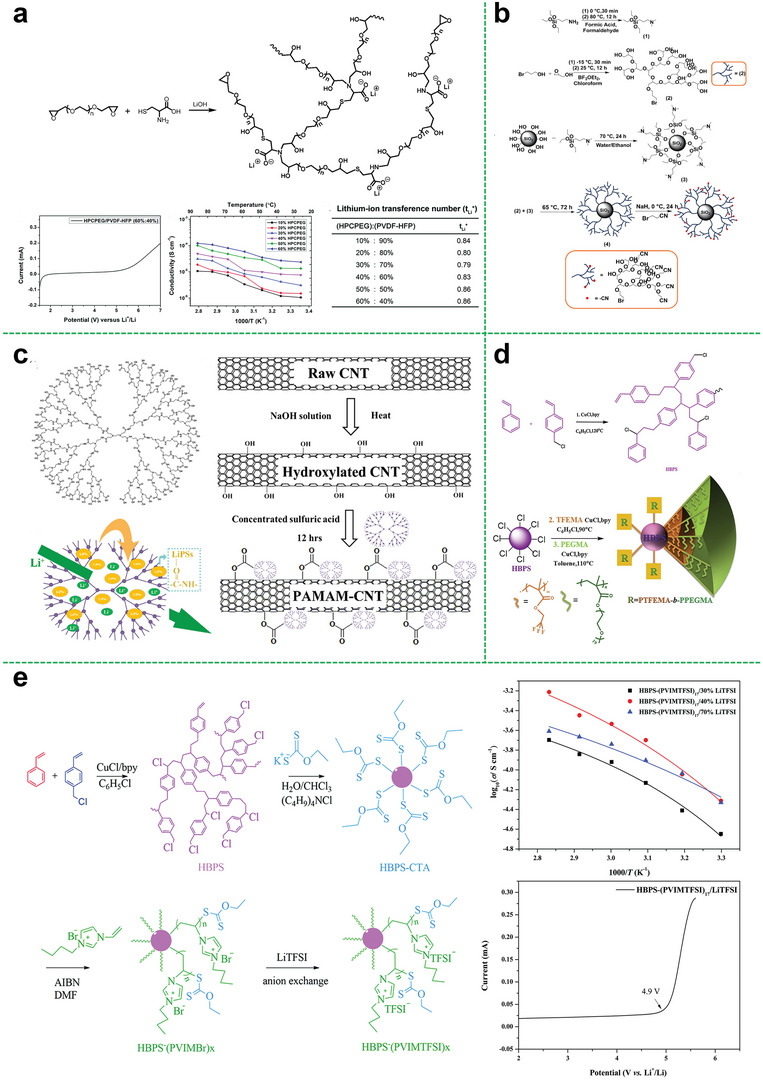
a) Synthesis of hyperbranched polyanionic HPCPEG; linear sweep voltammetry obtained for SIPE with an HPCPEG content of 60% at 25 °C; temperature dependence of ionic conductivity of HPCPEG/PVDF‐HFP SIPEs; lithium‐ion transference number of SIPEs with different HPCPEG contents at 25 °C. Reproduced with permission.^[^ ^49^ ^]^ Copyright 2019, The Royal Society of Chemistry. b) Synthesis of hyperbranched polyglycidol capped silica nanoparticles. Reproduced with permission.^[^ ^51^ ^]^ Copyright 2020, Wiley‐VCH GmbH. c) Chemical structure of PAMAM, illustration of lithium polysulfide adsorption and Li^+^ transport through PAMAM, and schematic illustration of PAMAM grafting on CNTs via esterification. Reproduced with permission.^[^ ^52^ ^]^ Copyright 2020, American Chemical Society. d) The synthetic route for fluorine‐containing star‐branched polymer. Reproduced with permission.^[^ ^55^ ^]^ Copyright 2018, Elsevier Ltd. e) The synthetic route of hyperbranched star‐shaped polymeric ionic liquids; the ionic conductivity measured (point) at different temperatures and the fitting results (line) by VTF equation; the electrochemical window of the HBPS‐(PVIMTFSI)_17_/LiTFSI electrolyte. Reproduced with permission.^[^ ^10^ ^]^ Copyright 2017, The Royal Society of Chemistry.

The various types of hyperbranched polymer electrolytes described above can also be referred to as dual ion conductors. Although with decades of development, this type of polymer electrolyte has been greatly enhanced in some properties, the further improvement of the ion transference number is still an enormous challenge. In a dual ion conductor electrolyte system, anions and cations conduct electricity at the same time, resulting in the low lithium ion transference number. In this regard, single‐ion polymer electrolytes (SIPEs) are developed as a solution to promote the migration of lithium ions and avoid the concentration polarization caused by anions converge at the electrolyte‐electrode interface. Specifically, anions like carboxylates, sulphonates, or trifluoromethyl‐sulfonylimide (TFSI) are covalently bonded to polymer backbones to keep the Li^+^ as the only mobile species.^[^ ^44^, ^45^, ^46^, ^47^, ^48^ ^]^ Therefore, the ion transference number can be enhanced. Zhang et al. first designed a hyperbranched poly (cysteine‐*co*‐poly (ethylene glycol) diglycidyl ether).^[^ ^49^ ^]^ As shown in **Figure** **4**a, the SIPEs were prepared by the obtained hyperbranched polymer and PVDF‐HFP. Owing to the hyperbranched structure and the introduction of the single‐ion system, the obtained SIPEs exhibited a high ionic conductivity of 1.2 × 10^−4^ S cm^−1^ at 85 °C, a high lithium ion transference number of 0.86 and a stable voltage window up to 4.8 V. Moreover, the assembled battery exhibited a specific capacity of 141 mA h g^−1^ at 0.1 C, and maintained ≈93% over 10 cycles with the retention value of coulombic efficiency up to about 94%, indicating good cycling stability.

The electrolytes compositing the polymer with polymer may have a problem of low mechanical strength, and cross‐linking is a good solution for improving mechanical property of polymer electrolyte. Zhang fabricated a thiol‐branched all‐solid‐state polymer electrolyte (SPE), which had high ionic conductivity and good film‐forming ability.^[^ ^50^ ^]^ First, they synthesized hyperbranched poly(glycidol) (HPG) by anionic polymerization using 2‐ethyl‐2(hydroxymethyl)‐1,3‐propanediol. Then, HPG and mercaptoacetic acid formed HPG‐SH (HPG (chemical decorated by —SH) via esterification. Finally, the polymer electrolyte was prepared by photopolymerization after mixing the HPG‐SH and trimethylolpropane propoxylate triacrylate (TPPTA). The film formation and film strength were adjusted by changing the ratio of raw materials. Besides, the ionic conductivity of the synthesized electrolyte was increased to 1.09 × 10^−4^ S cm^−1^ at 40 °C. The ion transference number of the obtained electrolyte could also be up to 0.31.

#### 2.2.3 Polymer‐Inorganic Materials

The hyper‐branched polymer can also composite with inorganic materials as many inorganic substances. Commonly, the introduction of inorganic fillers can effectively improve the mechanical strength, ionic conductivity, electrochemical stability of the polymer electrolyte. To effectively enhance the comprehensive performance of the electrolyte, it is a key to solve the compatibility of the polymer electrolyte matrix and inorganic filler. The surface of the inorganic filler is modified with polymer to make it more compatible with the polymer matrix. Mallela et al. used silica nanoparticles (SiO_2_) grafted with hyperbranched polyglycidol to prepare the composite solid polymer electrolyte (Figure 4b), improving the ionic conductivity, electrochemical stability, and dimensional stability of the electrolyte due to the introduction of silica.^[^ ^51^ ^]^ Besides silica, the carbon nanotubes are also effective additive. Li et al. grafted hyperbranched poly(amidoamine) on hydroxylated carbon nanotubes (PAMAM‐CNTs) as multifunctional interlayer for lithium batteries (Figure 4c).^[^ ^52^ ^]^ The assembled lithium‐ion battery has superior cycle performance, high energy density, and energy efficiency. The branched structure and the presence of carbon nanotubes make it also very useful for suppressing the shuttle effect in lithium‐sulfur batteries, the assembled battery displays a low capacity fading rate of 0.037% per cycle over 1200 cycles at 2 C.

In summary, hyperbranched polymers have been proved to be one of the promising hosts for the preparation of solid‐state polymer electrolytes due to its unique structural characteristics such as high segmental motion ability, amorphous state. Furthermore, the large number of terminal groups in hyperbranched polymers can be further modified to improve the mechanical properties and ion conductivity. Therefore, the introduction of hyperbranched polymers into polymer electrolyte system is beneficial to the promotion of performance. Noteworthily, the single‐component hyperbranched polymer is very limited in improving the ionic conductivity, and the composite system seems to be a more effective strategy. With careful selection of components into the composite electrolyte system, promoted the comprehensive performance of the polymer electrolytes can be achieved. Importantly, how to adjust the ratio of each component in the composite system to achieve the best performance is the key.

## 3 Star Polymer Electrolytes

Star polymers are an important type of polymer with topological structure. The star polymer can be formed by connecting the multiple linear polymer chains to a single core through chemical bonds. Particularly, star polymers can also be used as the polymer electrolyte matrix because of its spherical symmetrical topological structure, less chain entanglement, and low crystallinity. Compared with the traditional linear PEO‐based electrolyte, the crystallinity of the polymer matrix can be effectively restricted by the topological structure of the star‐shaped polymer. And the increased amorphous fraction and mobility of segments facilitate ion transport, and a large number of functional groups can be introduced into arms or core of the star polymer matrix, which is conducive to dissociation of lithium salt. Hence, the ionic conductivity at ambient temperature can be greatly improved. Moreover, the star‐shaped polymer can also improve the compatibility with the electrode surface to some extent due to its flexible polymer scaffold. Therefore, star‐shaped polymers are desirable electrolyte matrix materials for solid‐state batteries.

### 3.1 Star Polymer Electrolytes Based on Different Core

The star polymer is an ideal candidate of polymer electrolyte matrix due to its unique structural characteristics. According to the different core of star polymer electrolyte matrices, it can be divided into the following categories.

#### 3.1.1 Polystyrene Core

Although polystyrene (PS) does not have the capability of ion conduction, it can provide a rigid skeleton and increase the mechanical strength of the polymer electrolyte membranes. Besides, the introduction of fluorine element in the polymer matrix could give it various improvements including good flexibility, high mechanical strength, good water resistance, electrochemical stability, and high‐temperature resistant performance.^[^ ^53^, ^54^ ^]^ Xu et al. synthesized the fluorine‐containing star‐branched polymer in the structure of HBPS‐(PTFEMA‐*b*‐PPEGMA)_27_ by atom transfer radical polymerization (ATRP) (Figure 4d).^[^ ^55^ ^]^ Owing to the branched star‐shaped structure and the introduction of fluorine element, the prepared polymer electrolyte could exhibit good mechanical strength and electrochemical properties. The ionic conductivity was increased to 2.4 × 10^−5^ S cm^−1^ at 25 °C and the lithium‐ion transference number could reach 0.26 at 60 °C. And the obtained electrolyte possessed a wide electrochemical window (4.9 V), indicating the good comprehensive performances of fluorine‐containing star polymer electrolytes.

To further optimize electrolyte performances, Wang et al. provided an effective approach to synthesize a hyperbranched star‐shaped polymeric ionic liquid (Figure 4e).^[^ ^10^ ^]^ The electrolyte membranes exhibited great film‐forming property, which is not only conducive to the industrial processing and assembly but also beneficial to improving the safety of lithium‐ion batteries. A room‐temperature ionic conductivity of the hyperbranched star‐shaped polymeric ionic liquid was increased to 4.76 × 10^−5^ S cm^−1^. The electrochemical window of the obtained electrolyte could be up to 4.9 V. However, the relatively high structural rigidity of the hyperbranched star‐shaped polymeric ionic liquid leads to high resistance, because of the undesirable contact with the electrode.

**Figure 5:**
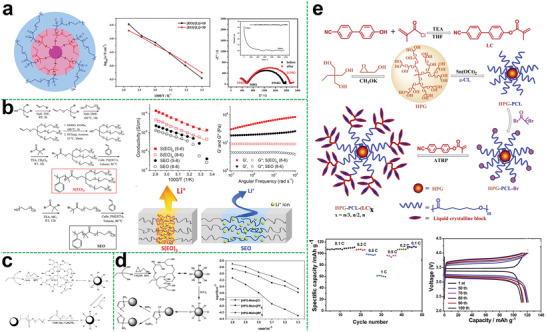
a) The structure of hyperbranched star polymer HBPS‐(PMMA‐bPPEGMA)*_x_*; temperature dependence of the conductivity of HBPS‐(PMMA‐*b*‐PPEGMA)_46_/LiTFSI (51.6% PPEGMA, [EO]/[Li]=30) with different ionic liquids; time‐dependence response of polarization and impedance spectra for HBPS‐(PMMA‐*b*‐PPEGMA)_46_ (51.6 wt% PPEGMA, [EO]/[Li]=30) electrolyte obtained at 60 °C and polarized with a potential of 10 mV. Reproduced with permission.^[^ ^56^ ^]^ Copyright 2016, Elsevier Ltd. b) Synthetic procedures for S(EO)_3_ miktoarm star copolymers; synthetic procedures for SEO linear diblock copolymers; temperature‐dependent ionic conductivity of S(EO)_3_ and SEO, solid lines represent the Arrhenius model fits the conductivity data; Storage (G′, filled symbols) and loss (G″, open symbols) moduli of S(EO)_3_ (8−6) and SEO (8−6) during frequency sweep at 40 °C and at a strain of 0.1%; schematic drawings of S(EO)_3_ and SEO electrolytes, depicting efficient lithium ion transport in (PEO)_3_ phases of S(EO)_3_ by confinement effects. Reprinted with permission.^[^ ^57^ ^]^ Copyright 2018, American Chemical Society. c) Synthesis route of hyperbranched star polymer HPG‐PPEGMA. Reproduced with permission.^[^ ^58^ ^]^ Copyright 2014, Springer. d) Synthesis route of ionic liquid polymers; temperature dependence of ionic conductivity of the polymer electrolytes [HPG‐MeIm]CI/LiTFSI, [HPG‐MeIm]BF_4_/LiTFSI and [HPG‐MeIm]PF_6_/LiTFSI. Reproduced with permission.^[^ ^59^ ^]^ Copyright 2014, Springer. e) Synthetic routes of LC monomer and HSLCPs; discharge performance at 0.1, 0.2, 0.5, and 1 C rate capability at 60 °C; charge–discharge curves at 0.1 C rate, 60 °C. Reproduced with permission.^[^ ^63^ ^]^ Copyright 2017, Elsevier Ltd.

In another work, Wang et al. introduced the PMMA‐*b*‐PPEGMA as ion conductive arms into the core of hyperbranched PS (HBPS) (**Figure** **5**a).^[^ ^56^ ^]^ The ionic conductivity of the electrolyte with a molar ratio of [EO]/[Li] of 30 could reach 8.3 × 10^−5^ S cm^−1^ at 30 °C. The lithium‐ion transference number of the prepared electrolyte could be up to 0.31. And the interface impedance had been reduced, revealing that the grafted chain segments of the hyperbranched star polymer electrolyte matrix could greatly affect the electrochemical properties of the electrolyte and interfacial contact between the electrolyte and electrode.

Importantly, mechanical strength of the polymer electrolyte is also an important parameter for practical application of the electrolyte membranes. The excellent mechanical strength can effectively avoid the puncture of lithium dendrites during battery cycling. In this regard, introduction of rigid segments into the star‐shaped polymer also has a unique effect on enhancing the mechanical strength for polymer electrolytes. As shown in Figure 5b, Miktoarm star copolymers composed of three poly (ethylene oxide) (PEO) arms connected to one PS chain were prepared by Lee et al.^[^ ^57^ ^]^ It was demonstrated that lithium‐ion transport along the (PEO)_3_ phases became noticeably efficient with improved storage moduli over a wide temperature window. Moreover, combining three PEO arms in one PS block, the crystallization of PEO was substantially reduced, thus the room temperature ionic conductivity of the electrolyte was improved by an order of magnitude compared with the linear one.

Generally, PS as the core for star‐shaped polymer electrolytes has excellent mechanical strength and good dimensional stability, which is promising for the enhancement of mechanical properties for polymer electrolytes systems. However, to achieve the balance of moderate mechanical qualities and exceptional ion mobility, the ratio of PS with other ion‐conducting components must be carefully tuned because the PS lacks the ability to conduct ions.

#### 3.1.2 Polyether Core

Hyperbranched polyether as the core for star polymer can provide more conductive segments to dissolve more lithium salts and a large number of terminal groups can be further modified to optimize performance. Zheng et al. first synthesized the star polymer electrolyte using hyperbranched poly(glycidol) as the core (Figure 5c).^[^ ^58^ ^]^ The HPG‐PPEGMA electrolyte exhibited ionic conductivity of 1 × 10^−4^ S cm^−1^ at 30 °C (6.8 × 10^−4^ S cm^−1^ at 80 °C) in the condition of [EO]/[Li] was 20, showing a significant promotion of ion conductivity compared with the linear one.

Typically, the ionic liquid is a liquid with all ionic components which possess many excellent characteristics such as low viscosity, nonvolatility, low toxicity, and recycling. The application of ionic liquids in the field of electrochemistry has greatly promoted the progress of polymer electrolytes. Introducing ionic liquids into star polymer electrolyte systems is also an effective way to improve electrolyte performances. Zheng et al. also reported a series of HPG‐based ionic liquid polymers.^[^ ^59^ ^]^ The ionic conductivity of obtained electrolytes could each be 3.5 × 10^−4^ S cm^−1^ at 30 °C (Figure 5d).

Thus, the components with electrochemical properties can be introduced in the star polymer system to achieve higher ionic conductivity at room temperature. And these findings contribute in several ways to our understanding of molecular structure designing and choice of materials, meanwhile, providing a basis for the development of new materials.

Recently, one of the most promising methods to further improve the performance of hyperbranched star polymer electrolytes is to introduce liquid crystals (LCs) into the polymer electrolytes. It has been reported that polymers modified by functional liquid crystals can construct ion‐conductive channels, which can effectively improve the ionic conductivity of polymer electrolyte.^[^ ^60^, ^61^, ^62^ ^]^ Wang et al. prepared a novel hyperbranched star liquid crystal polymer (HSLCP) electrolyte (Figure 5e).^[^ ^63^ ^]^ Owing to the design of the branching structure and the orientation of liquid crystal segments, the obtained HSLCP electrolyte exhibits good electrochemical properties such as high ionic conductivity (5.98 × 10^−5^ S cm^−1^ at 30 °C), outstanding ion transference number (0.63), and wide electrochemical window (5.12 V). And the assembled LiFePO_4_‐based battery showed an initial discharge capacity of 114 mAh g^−1^ and then kept at 112 mAh g^−1^ after 100 cycles at 60 °C, resulting in 98% of capacity retention, and the battery also performed good in rate testing, as shown in Figure 5e. Visibly, the introduction of liquid crystal with ordered structure is indeed beneficial to improving the electrochemical and mechanical properties of the battery system. This provides a reference for the subsequent structural design.

**Figure 6:**
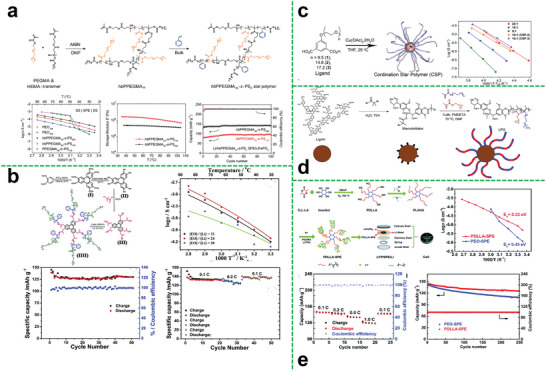
a) Synthesis of hbPPEGMA*_m_*‐*s*‐PS*_n_* hyperstar polymers; temperature‐dependent ionic conductivity of hbPPEGMA*_m_*‐*s*‐PS*_n_* SPEs; storage modulus E′; cycling performance of Li/hbPPEGMA*_m_*‐*s*‐PS*_n_* SPE/LiFePO_4_ cells at 0.2 C, 60 °C. Reproduced with permission.^[^ ^64^ ^]^ Copyright 2019, American Chemical Society. b) Synthetic route for DLC‐((PS)_23_‐*b*‐(PPEGMA)_10_)_6_; temperature dependence of the ionic conductivity of DLC‐((PS)_23_‐*b*‐(PPEGMA)_10_)_6_/LiTFSI at various [EO]/[Li]; cycle performance of the LFP/Li cell at 0.1 C; specific capacity of the battery at the current densities of 0.1 and 0.2 C. Reproduced with permission.^[^ ^65^ ^]^ Copyright 2018, Elsevier B.V. c) Schematic of the synthesis of CSPs via convergent route; conductivity as a function of inverse of temperature for CSP‐1; 20:1 (red), 10:1 (blue), 5:1 (olive), CSP‐2 10:1 (orange), CSP‐3 10:1 (violet). Solid lines are respective fits obtained using VFT expression. Reproduced with permission.^[^ ^69^ ^]^ Copyright 2018, American Chemical Society. d) Synthetic routes for lignin‐derived graft polymer with ion‐conducting PEGMA and cross‐linkable GMA moieties (LPG), where the PEGMA/GMA molar ratio is 85:15. Reproduced with permission.^[^ ^8^ ^]^ Copyright 2020, Wiley‐VCH. e) Schematic illustration for the preparation process of PLAHA, PDLLA‐SPEs, and the cell; Arrhenius plots for SPEs with the molar ratio of EO/Li^+^ =16:1; the rate performance of LFP/PDLLA‐SPE/Li battery with the molar ratio of EO/Li^+^ =16:1 at 60 °C; the cycling performance of PDLLA‐SPE and PEO‐SPE with the molar ratio of EO/Li^+^ =16:1 at 60 °C at the current density of 0.2 C. Reproduced with permission.^[^ ^76^ ^]^ Copyright 2018, The Royal Society of Chemistry.

In another work, Chen et al. developed a hyper star polymer electrolyte with hyperbranched PEO serving as the star core and linear PS serving as the arms (**Figure** **6**a).^[^ ^64^ ^]^ The hyperbranched PEO (PPEGMA) core can suppress crystallinity and thus enhances the ionic conduction, meanwhile, the PS arms can provide mechanical strength. Thus, the obtained hyper star polymer electrolyte could exhibit a desirable ionic conductivity and a good storage modulus. Furthermore, the assembled battery also delivered a greater performance.

Generally, the star polymer electrolytes with hyperbranched polyether as the core could effectively inhibit the crystallization of the polymer matrix and provide more amorphous regions for ion conduction. However, its mechanical strength is not enough for using directly, crosslinking monomers or other rigid units should be introduced into the system to further improve the mechanical properties of the electrolyte.

#### 3.1.3 Liquid Crystals Core

As reported, liquid crystals with ordering properties are appealing substance to construct ordered ion channels for star‐shaped polymer electrolytes. Wang et al. designed a controlled‐structure discotic liquid crystal (DLC)‐based six‐arm star copolymer electrolyte (Figure 6b).^[^ ^65^ ^]^ The introduction of the discotic liquid crystal with ordered structure resulted in even higher Li‐ion diffusion mobility. Ionic conductivity of this electrolyte system was 1.46 × 10^−4^ S cm^−1^ at 30 °C, which was more than eight times higher than that of the corresponding linear copolymer electrolytes, and the assembled battery also has excellent performance. Especially, this work reveals the new role of liquid crystals in ion conduction and also guides the selection of new polymer electrolyte materials. The liquid crystal not only provides mechanical strength, but also can build transmission channels for promoting ion conduction.

#### 3.1.4 Metal‐Organic Polyhedra Core

Coordination star polymers (CSPs) are a new class of inorganic‐organic hybrid star‐shaped molecules that possess discrete metal‐organic polyhedra (MOP) core decorated with radiating organic polymer arms.^[^ ^66^, ^67^, ^68^ ^]^ It was reported that CSPs are a unique class of compound, with synergistically combined advantages of metal‐organic materials and polymers. Nagarkar et al. reported the modulation of self‐assembled microstructure and dynamics of CSP materials and its utilization for ion transport (Figure 6c).^[^ ^69^ ^]^ The assembled structure of CSPs exhibited glassy nature with short‐range structural order while long‐range disorder with dynamic polymer arm domains. Besides, it was found that glassy CSPs with a dynamic polymer domain could be employed as the matrix for lithium‐ion conduction. The Li‐ion conductivity of such optimized glass materials could be demonstrated to be comparable to the widely used organic polymer electrolytes. Also, the ion conductivity of the obtained electrolyte can reach 1.1 × 10^−5^ S cm^−1^ at 60 °C, and larger the molecular weight is, higher the ionic conductivity is. The maximum ionic conductivity can increase to 3.7 × 10^−5^ S cm^−1^ at 60 °C.

Thus, CSPs are the new media for ion transportation and the potential candidates for desirable solid‐state electrolytes.

#### 3.1.5 Polyester Core

The polyester core can provide the polymer electrolyte with good flexibility, and electrochemical properties. Inspired by the superior electrochemical features of imidazolium and the superior feature of branched star‐shaped polymers, Zhou et al. prepared the four‐armed and imidazolium cation‐tethered polymeric ionic liquid (IMFPIL) through ATRP of hydroxyethyl acrylate and ion exchange.^[^ ^70^ ^]^ The branching architecture of the star IMFPIL could ensure the IMFPIL to possess high thermal stability with the maximum decomposition temperature up to 400 °C and a strong mechanical strength with fracture stress of 0.92 MPa at a maximal strain of 245.6% due to the introduction of ionic liquids. The enhanced maximum ionic conductivity of the electrolyte could reach 0.49 mS cm^−1^ at 60 °C, which was 22 times that of the linear counterpart with the same composition. Due to the good combination of ionic liquid and polyester skeleton, the assembled Li/LiFePO_4_ type cell presented a high discharge capacity of 153 mAh g^−1^ at 0.2 C with 99% of coulombic efficiency.

#### 3.1.6 Silsesquioxane Core

Polysiloxane with good dimensional thermal stability and low glass transition temperature is introduced into the structure of the polymer electrolyte matrix, the electrolyte can be easier to achieve higher safety and ion conduction at room temperature. Zhang et al. chose the octavinyl octasilsesquioxane (OV‐POSS) as the core,^[^ ^71^ ^]^ which can improve ion conductivity and enhance mechanical strength owing to its well‐defined nanoscale organic/inorganic hybrid structure.^[^ ^72^, ^73^, ^74^, ^75^ ^]^ And grafted the boron moieties (B‐PEGMA) and poly(ethyleneglycol)methyl ether methacrylate (PEGMEM) to the polyhedral oligomeric silsesquioxane (POSS) by free radical polymerization method. The star‐shaped POSS‐*g*‐PEGMEM/B‐PEGMA electrolyte with the boron moieties could further promote lithium salt dissociation. Thus, a high ionic conductivity of 3.44 × 10^−4^ S cm^−1^ and a high Li‐ion transference number of 0.58 at 25 °C were obtained. Furthermore, the coin cells of LFP/Li had great stability at room temperature, ether at 60 °C. Besides, the good rate performance could be also achieved.

#### 3.1.7 Lignin Core

The lignin is a kind of natural resource, and the multiple aromatic rings provide high thermal stability and mechanical strength. Besides, it has plenty of hydroxy groups for easy modification to fit for demand. Jeong et al. prepare the star‐shaped polymer electrolytes by controlled radical polymerization with lignin as the core, and the polymer chain segments on the arm include an ion conductive part and a provided cross‐linked part (Figure 6d).^[^ ^8^ ^]^ All these make the electrolyte membrane with excellent ionic conductivity, mechanical integrity, lithium dendrite suppression, and great long‐term cyclability of the assembled battery. The ionic conductivity of the electrolyte could reach up to 3.3 × 10^−5^ S cm^−1^ at 30 °C. The assembled LiFePO_4_‐based battery could maintain the capacity more than 150 mAh g^−1^ after 50 cycles at 60 °C in 0.1 C, and kept the capacity over 140 mAh g^−1^ with 100 cycles at 60 °C in 1 C and the capacity retention of the cell reached 99.2 %.

#### 3.1.8 Polylactide Core

Polylactide is another potential host for star‐shaped polymer electrolytes. Wang et al. prepared a novel star‐comb copolymer electrolyte based on poly(d, l‐lactide) (PDLLA) and poly (ethylene glycol)methyl ether methacrylate (PEGMA).^[^ ^76^ ^]^ The electrolyte had a wide electrochemical stability window up to 5.1 V, a good ionic conductivity, rate performance, and long cycle performance (Figure 6e).

#### 3.1.9 Cyclodextrin Core

**Figure 7:**
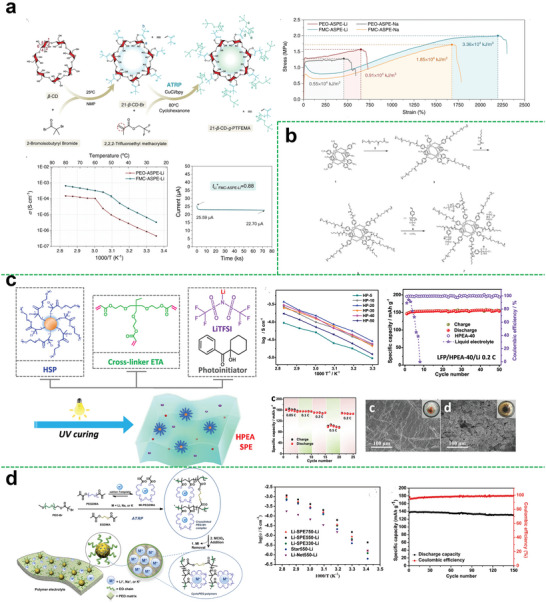
a) Detailed synthetic scheme of 21‐*β*‐CD‐*g*‐PTFEMA; ionic conductivity‐temperature functions of FMCASPE‐Li vs. PEO‐ASPE‐Li; polarization (POL) result of FMCASPE‐Li; the extension comparison of the four membranes. Reproduced with permission.^[^ ^77^ ^]^ Copyright 2022, The Royal Society of Chemistry. b) Synthesis of a star‐shaped single lithium‐ion conducting block copolymer with POSS nanoparticle as core. Reproduced with permission.^[^ ^80^ ^]^ Copyright 2017, Elsevier Ltd. c) Schematic illustration of the preparation of the HPEA SPE; temperature‐dependent ionic conductivities of HPEA‐based films with different content of cross‐linkers; specific capacity at different current densities of LFP/HPEA‐40/Li cell; cycle performance for LFP/HPEA‐40/Li cell with LFP/liquid electrolyte/Li cell as a comparison at 0.2 C; plane‐view SEM images of cycled Li electrode over 200 cycles from Li/HPEA‐40/Li and Li/liquid electrolyte/Li. Reproduced with permission.^[^ ^81^ ^]^ Copyright 2019, American Chemical Society. d) Schematic illustration on the star polymer with pseudo‐crown ether cavities and PEO arms; temperature‐dependent ionic conductivities of the tailor‐made star polymer electrolyte for lithium‐ion batteries; cycle performance of Li|K‐SPE750‐Li|LFP cell with a certain current density of 0.1 C at 60 °C. Reproduced with permission.^[^ ^82^ ^]^ Copyright 2019, Elsevier B.V.

Yun Su et al. designed a fluorine‐rich macromolecule‐containing all‐solid‐state polymer electrolyte (FMC‐ASPE), which involved 21‐arm fluoropolymers (21‐*β*‐CD‐*g*‐PTFEMA) with a postmodified *β*‐cyclodextrin (*β*‐CD) core and 21 poly(2,2,2‐trifluoroethyl methacrylate) (PTFEMA) arms, as shown in **Figure** **7**a.^[^ ^77^ ^]^ The star‐shaped polymer electrolyte is synthesized through ATRP. According to the results, the electrolyte has higher voltage stability, transference number (*t*_Li_^+^ = 0.88), and high ionic conductivity, that could reach to 6.43 × 10^−4^ S cm^−1^ at 80 °C for FMC‐ASPE‐Li. Besides, the fabricated electrolyte performed great on mechanical strength and thermal stability, the toughness of FMC‐ASPE‐Li was 3.7 times larger than that of the control group PEO‐ASPE‐Li.

In a word, both the core and the arms of the star‐shaped topological polymer electrolyte matrix have good designed ability, which is conducive to improving the comprehensive performance of the electrolyte by choosing appropriate functional components into the star‐shaped structure.

### 3.2 Composite Star Polymer Electrolytes

Sometimes, when star‐shaped polymer electrolytes cannot meet all the requirements of high‐performance electrolytes by adjusting its structure, it is an effective method of blending other organic or inorganic components with star polymer electrolyte to achieve a great improvement in performance of the electrolyte.

#### 3.2.1 Polymer‐Polymer

Be distinguished from the traditional dual ion conductors, the single‐ion conducting polymer electrolytes (SCPEs) are well recognized for increased energy efficiency and ion transference number because of their capability to mitigate electrode polarization and lower electrolyte loss.^[^ ^44^, ^45^, ^78^, ^79^ ^]^ Like hyperbranched polymer electrolytes, the single‐ion conductive polymer segments can also be introduced to the structure of the star polymer electrolyte matrix. Cao et al. first synthesized an innovative SCPE using POSS as the core then grafting the carboxylic acid terminated PEO and the single‐ion conductive unit STF‐Li^+^ onto POSS nanoparticle (Figure 7b).^[^ ^80^ ^]^ The SSCP‐Li^+^ with the incorporation of the POSS nanoparticles could exhibit enhanced thermo‐stability with no significant weight loss up to 300 °C. And the ionic conductivity could achieve 1.05 × 10^−5^ S cm^−1^ at 60 °C by adding extra PEO to make a polymer blend with the [EO]/[Li^+^] at 20.

Chen et al. made the modified hyperbranched star polymer (HBPS‐PPEGMA) embedded into a 3D cross‐linking network through one‐step photopolymerization (Figure 7c).^[^ ^81^ ^]^ Due to the enhancement of mechanical behavior and the promotion of polymer chain segmental dynamics in the 3D cross‐linking structure, the prepared SPE showed improved ionic conductivity, wide electrochemical stability window of 5.1 V, enough mechanical strength to suppress the lithium dendrite, and good cell performances.

In another aspect, the structure of precisely imprinted polymeric pseudo‐crown ether cavities was reported to prepare a star‐shaped polymer electrolyte. A tailor‐made star polymer comprised of cross‐linked cores containing polymeric pseudo‐crown ether cavities and linear PEO arms was designed by Xiao et al. (Figure 7d).^[^ ^82^ ^]^ Owing to the coordination between the pseudo‐crown ether cavities in cross‐linked cores and alkali metal cations, more cation diffusion channels would be offered to enhance the ion mobility. Thus, the ionic conductivity of the electrolyte could reach 1.48 × 10^−5^ S cm^−1^ at 20 °C. A wider electrochemical window of 5.3 V at 25 °C could be achieved. Also, the assembled cell exhibited higher discharge capacities and capacity retention (Figure 7d).

Thus, it can be seen that the star‐shaped polymer‐polymer composite electrolyte system showed meliorative electrochemical properties because of the good compatibility between two or more polymer components. With the designing of 3D cross‐linked network or precisely imprinted polymeric pseudo‐crown ether cavities, the room temperature ionic conductivity and mechanical strength of electrolyte has been greatly improved.

#### 3.2.2 Polymer‐Plasticizer

**Figure 8:**
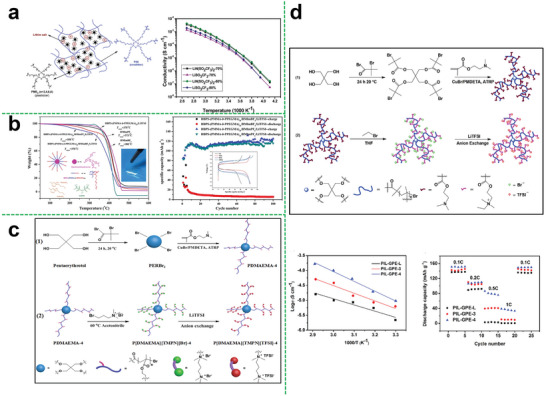
a) Schematic structure of semi‐IPN SPE; temperature dependence of ionic conductivities using LiSO_3_CF_3_ and LiN(SO_2_CF_3_)_2_ as lithium salts at [EO]/[Li^+^] = 15, respectively (PA6 with 70 or 80 wt% of PME_4_). Reproduced with permission.^[^ ^83^ ^]^ Copyright 2012, American Chemical Society. b) TGA curves of electrolytes; inset: photograph of HBPS‐(PMMA‐*b*‐PPEGMA)_30_/BMImPF_6_/LiTFSI polymer electrolytes with 30 wt% BMImPF_6_; cycle performance of Li/LiFePO_4_ cells based on HBPS‐(PMMA‐*b*‐PPEGMA)_30_/ionic liquid/LiTFSI electrolyte at 60 °C under the current density of 0.1 C; inset: the corresponding charge/discharge curves of the HBPS‐(PMMA‐*b*‐PPEGMA)_30_/BMImPF_6_/LiTFSI electrolyte based cell at different cycle numbers at 60 °C. Reproduced with permission.^[^ ^85^ ^]^ Copyright 2017, Springer. c) Synthetic procedure for dicationic star topological polymeric ionic liquid (DPIL‐4). Reproduced with permission.^[^ ^86^ ^]^ Copyright 2019, Elsevier B.V. d) Synthetic approach for star‐shaped four‐arm PIL‐4; temperature dependence of the ionic conductivity for PIL‐GPE‐L, PIL‐GPE‐3, and PIL‐GPE‐4; C‐rate capacities for LiFePO_4_/Li batteries assemble using PIL‐GPE‐L, PIL‐GPE‐3, and PIL‐GPE‐4. Reproduced with permission.^[^ ^87^ ^]^ Copyright 2019, Elsevier B.V.

In this regard, plasticizers are the desirable additives that can be used to modify the electrolytes. Specifically, plasticizers can not only increase the amorphous regions in the polymer matrix but also enhance the ability of polymer segment movement. He et al. prepared a semi‐interpenetrating network (semi‐IPN) using the star‐shaped cross‐linker (PA6) and the star‐shaped plasticizers (PME*_n_*) (**Figure** **8**a).^[^ ^83^ ^]^ Due to the introduction of star‐shaped plasticizers, the crystallization of oligo(ethyleneoxy) side groups was prevented efficiently. Thus, the maximum ionic conductivity of the obtained electrolyte was found to be 1.98 × 10^−4^ S cm^−1^. Especially, the electrolyte containing 80 wt% of PME_4_ had a wide electrochemical stability window of above 5.0 V. However, the calculated Coulombic efficiency of lithium plating/stripping was relatively low (below 80%), probably due to the undesirable compatibility between the electrolytes and lithium metal electrode.

In addition to semi‐interpenetrating networks, constructing cross‐linked co‐networks is also a common design strategy for improving performance of the electrolyte. In this regard, the star‐branched polymeric co‐networks consisting of a three‐arm poly (ethylene glycol) acrylate macromonomers (PEG3A) and a three‐arm polysulfide (PS) as well as the succinonitrile (SCN) plasticizer synthesized by UV photopolymerization were reported by Onozuka et al.^[^ ^84^ ^]^ Benefiting from the plasticizing effect of succinonitrile, the electrolyte showed a lower glass transition temperature (*T*_g_ = −49 °C), a high ion transference number of 0.7, and an ionic conductivity of 1.35 × 10^−3^ S cm^−1^ at ambient temperature with a tensile strength of 0.3 MPa.

Unlike grafting ionic liquids onto star‐shaped polymers, ionic liquids as the plasticizer can also be directly compounded with star‐shaped polymers to prepare the composite star‐shaped polymer electrolytes. Wang et al. designed a new composite polymer electrolyte composed of hyperbranched star polymers (HBPS‐(PMMA‐*b*‐PPEGMA)_30_ and the ionic liquids (BMImBF_4_ or BMImPF_6_) (Figure 8b).^[^ ^85^ ^]^ The room temperature ionic conductivity of the composite electrolytes with 40 wt% BMImBF_4_ or 40 wt% BMImPF_6_ could reach 2.5 × 10^−4^ and 4.1 × 10^−5^ S cm^−1^, respectively. This kind of method can simplify synthesis with guaranteed electrochemical and battery performance.

**Figure 9:**
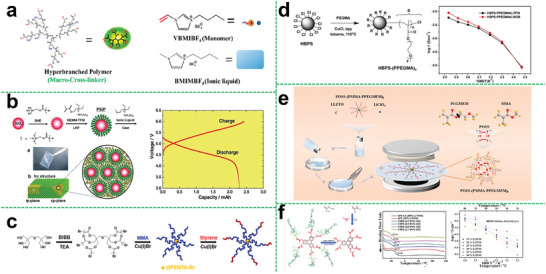
a) Schematic illustration for synthesis of PIL ionogel with hyperbranched polymer as macro‐cross‐linkers by photopolymerization. Reproduced with permission.^[^ ^88^ ^]^ Copyright 2021, American Chemical Society. b) Preparation process for novel PSiP/IL‐solid electrolyte with 3D assembling PSiPs, thereby forming network channel of high ion‐conductivity, photograph of produced solid film, and analyzed structure of PSiP array in solid; charge/discharge curve of a LIB using PSiP/IL solid electrolyte. Reproduced with permission.^[^ ^89^ ^]^ Copyright 2011, WILEY‐VCH. c) Synthetic scheme of star‐shaped block copolymers (MS)_6_. Reproduced with permission.^[^ ^90^ ^]^ Copyright 2019, American Chemical Society. d) Synthetic route of HBPS‐(PPEGMA)*_x_*; temperature dependence of conductivity for polymer electrolytes with different lithium salts. Reproduced with permission.^[^ ^94^ ^]^ Copyright 2014, Springer. e) Schematic diagram of preparation process of LP‐CPEM and synthesis of star‐shaped POSS−(PMMA−PPEGMEM)_8_. Reproduced with permission.^[^ ^26^ ^]^ Copyright 2021, American Chemical Society. f) Synthetic route of HHTP‐PMMA‐PPEGMA.HHTP, hexahydroxytriphenylene; PMMA‐PPEGMA, poly (methyl methacrylate)‐poly (poly [ethylene glycol] methyl ether methacrylate); DSC curves of the polymer electrolytes (same Li salt contents but different molecular weight of the polymer matrix) and the composite electrolytes with different GO content; Arrhenius plots of SPE at given content of LiTFSI. Reproduced with permission.^[^ ^96^ ^]^ Copyright 2020, Wiley Periodicals LLC.

Besides, Zhou et al. prepared a type of star‐shaped polymer electrolyte based on the novel dicationic tetraalkylammonium‐based polymeric ionic liquid and another ionic liquid plasticizer (DEME‐TFSI) (Figure 8c).^[^ ^86^ ^]^ Due to the more amorphous region and higher mobility of polymer segments after introducing the ionic liquid, the ionic conductivity of the obtained electrolyte could achieve 0.114 mS cm^−1^ at 30 °C, which was 42.5 times that of the linear counterpart. Moreover, the assembled LFP‐based battery exhibited excellent rate performance due to the meliorated electrochemical characteristics of the prepared electrolytes. In their other work, an analogous star‐shaped polymeric ionic liquids based on poly(2‐(dimethyl‐ethyl‐amino)ethyl methacrylate) bis(trifluoromethylsulfonyl)imide was prepared through ATRP (Figure 8d).^[^ ^87^ ^]^ By adding an ionic liquid plasticizer (DEME‐TFSI), the comprehensive performances of the GPE could be superior to the linear contrastive sample. Also, Wang et al. have an analogous work combining the poly(ionic liquid) and hyperbranched polymer with a 3D printable single network(**Figure** **9**a), obtaining polymer electrolyte with high stretchability, superior ambient temperature ionic conductivity which could reach 5.8 mS cm^−1^, and thermal stability.^[^ ^88^ ^]^ Sato et al. designed a polymer electrolyte based on colloidal crystal of hybrid silica particles (PSiPs) and ionic liquid polymer brushes (Figure 9b).^[^ ^89^ ^]^ Owing to the ability to construct a continuous channel in face‐centered cubic (fcc) structure, the modified particles showed an improved ionic conductivity, which could reach 0.17 mS cm^−1^ at 30 °C. Besides, the discharge specific capacity and columbic efficiency of the Li_4_Ti_5_O_12_/LiMn_2_O_4_ type bipolar cell were 2.3 mAh and 95% at the fifth cycle, respectively.

All of the above works show that ionic liquids are a kind of efficient plasticizers in improving the electrochemical properties.

To improve the mechanical strength of ionic gel polymer electrolyte system, Hwang et al. designed a six‐arm star‐shaped block copolymer based on (poly(methyl methacrylate)‐*b*‐polystyr‐ene)_6_ ((MS)_6_) and an ionic liquid of 1‐ethyl‐3‐methylimida‐zolium bis(trifluoromethyl sulfonyl)imide ([EMI][TFSI]) (Figure 9c).^[^ ^90^ ^]^ The (MS)_6_ had more than twice the mechanical modulus compared to linear polystyrene‐*b*‐poly (methyl methacrylate)‐*b‐*polystyrene (SMS), while maintaining high ionic conductivity (≈1.54 mS cm^−1^) at room temperature.

Taken together, these results suggest that the well‐defined structure of electrolyte and the addition of an appropriate amount of plasticizer are especially important to the performance of the electrolyte, and can provide an effective route for the enhancement of electrochemical properties by the introduction of plasticizers with special functions in the star polymer electrolyte system. However, it is worth noting that the introduction of an excess amount of organic small molecular plasticizer can cause irreversible damage to the mechanical strength of the electrolyte membranes, which may trigger the potential safety hazards. Thus, it is essential to explore the optimal mixing ratio between the polymer matrix and the plasticizers for realizing the best performance of the electrolyte membranes.

#### 3.2.3 Polymer‐Inorganic Materials/Nanomaterials

The inorganic nanomaterials as additives are not only conductive to improving the mechanical behaviors of electrolytes but also facilitate to enhance the electrochemical performances.^[^ ^91^, ^92^, ^93^ ^]^ In this regard, the hyperbranched star polymer HBPS‐(PPEGMA)*_x_* was first synthesized by ATRP and then the nano‐TiO_2_ was added to the polymer matrix to prepare the composite polymer electrolyte by Ren et al.^[^ ^94^ ^]^ It was shown that the ionic conductivity of the composite polymer electrolyte was notable improved when TiO_2_ content was added and the size of TiO_2_ was 20 nm (Figure 9d). But one of the major drawbacks to adopting using this system is that the content of nano‐fillers (particle agglomeration caused by the higher concentration of nanoparticles) will largely limit the performance of obtained electrolytes. Therefore, the size and content of the fillers and the compatibility between polymer electrolyte matrix and nanomaterials need to be selected carefully, which would have a dramatic impact on performance.

Besides, Ma et al. synthesize an eight‐armed star‐shaped polymer of octa(poly(methyl methacrylate)−poly(poly(ethylene glycol) methyl ether methacrylate) cageoligomeric silsesquioxanes (POSS−(PMMA−PPEGMEM)_8_) that synthesized by one‐step free ATRP, then, they obtained the hybrid polymer electrolyte membrane (LP‐CPEM) by combining the eight‐armed star‐shaped polymer with Li_6.4_La_3_Zr_1.4_Ta_0.6_O_12_ (LLZTO) (Figure 9e).^[^ ^26^ ^]^ Beneficial from large number of transport chain segments and the incorporation of inorganic materials, the composite electrolyte system formed a continuous ion migration channel, high ion conductivity (3.8 × 10^−4^ S cm^−1^ at 30 °C), and wide electrochemical stabilization window of 5.2 V were obtained.

Similarly, a small amount of carbon nano‐materials may also have the ability to improve the electrochemical performances of composite polymer electrolytes. Wang et al. attempted in this regard. They introduced the carbon nano‐tube (CNT) and fullerene (C_60_) into the hyperbranched star polymer (HBPS‐(PMMA‐*b*‐PPEGMA)_30_) matrix.^[^ ^95^ ^]^ And the ionic conductivity of the HBPS‐(PMMA‐*b*‐PPEGMA)_30_/CNT/LiTFSI electrolyte with 0.2 wt% CNT could reach 1.06 × 10^−5^ S cm^−1^ at 30 °C to the utmost extent. As for lithium‐ion transference numbers, the CNT‐based electrolyte was raised to 0.52, probably due to the fast migration of lithium ions in the desirable interfacial layers between carbon nanomaterials and polymer matrix. And the addition of inorganic fillers facilitates battery cycle testing. Besides, the Graphene‐Oxide (GO) is also a kind of nano‐fillers that could improve the electrochemical and mechanical properties because of the high oxidation, high Young's modulus, and high aspect ratio. Zhang et al. used triphenylene as core and block poly(methyl methacrylate)‐poly(poly[ethylene glycol] methyl ether methactylate) as arm synthesized a novel six arm star polymer matrix (Figure 9f).^[^ ^96^ ^]^ After mixing the GO with polymer electrolyte matrix, a flexible composite all‐solid‐state polymer electrolytes (CSPEs) was obtained via solution casting. The ionic conductivity at ambient temperature of CSPEs could reach 3.69 × 10^−5^ S cm^−1^. In addition, the CSPE has better mechanical properties and thermal stability due to the presence of triphenylene and GO nano‐filler in the electrolyte system. It also has a wide electrochemical window of 4.9 V.

**Figure 10:**
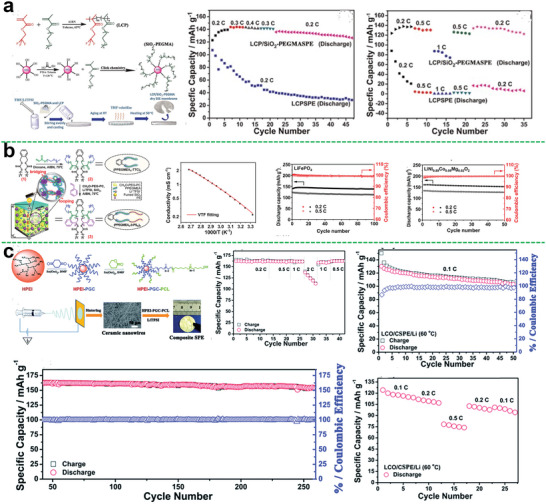
a) Synthesize routes of the LCP and the SiO_2_‐PEGMA and schematic illustration for the preparation of the LCP/SiO_2_‐PEGMA all‐solid‐state electrolyte membrane (LCP/SiO_2_‐PEGMASPE); cycling performances of the Li/LCP/LFP (at 0.2 C) and the Li/LCP/SiO_2_‐PEGMASPE/LFP cells (at 0.2, 0.3, 0.4 C); specific capacity of the LFP/Li cells at varied current density. Reproduced with permission.^[^ ^101^ ^]^ Copyright 2019, Wiley‐VCH. b) Schematic synthesis of SBBCEs; temperature‐dependent ionic conductivity of SBBCE‐2, the plots representing the experimental data, and the solid line representing VTF fitting results; charge and discharge curves of LiFePO_4_|SBBCE‐2|Li cells at 0.2 and 0.5 C at 28 °C; charge and discharge curves of LiNi _0.89_Co_0.09_Mg_0.02_O_2_|SBBCE‐2|Li cells at 0.2 and 0.5 C at 28 °C. Reproduced with permission.^[^ ^102^ ^]^ Copyright 2019, American Chemical Society. c) Schematic diagram of the synthesis route of the all solid‐state hyperbranched polymer matrix (HPEI–PGC–PCL) and the schematic illustration of the preparation process of the ceramic film containing ceramic nanowires and the CSPE; cycling performance of the all‐solid‐state LFP/Li cell using the CSPE film at C‐rates from 0.2 to 2 C; long‐term cycling performance of the LFP/CSPE/Li cell at 0.5 C after the high rate cycling; cycling performance of the LCO/Li cell using the CSPE film at 0.1 C; cycling performance of the LCO/Li cell using the CSPE film at the C‐rates of 0.1, 0.2 and 0.5 C. Reproduced with permission.^[^ ^103^ ^]^ Copyright 2019, The Royal Society of Chemistry.

Likewise, inorganic nano‐fillers such as SiO_2_, Al_2_O_3,_ and Fe_2_O_3_ are all common additives that can be used in composite polymer electrolytes.^[^ ^97^, ^98^, ^99^, ^100^ ^]^ On one hand, these inorganic nano‐fillers can decrease the crystallinity of the polymer matrix for increasing ionic conductivity. On the other hand, the introduction of the nano‐fillers is capable of enhancing the mechanical properties of electrolytes membranes. Wang et al. reported an inorganic‐polymeric composite electrolyte (**Figure** **10**a) composed of modified nano‐silica that grafted with flexible poly (ethylene glycol) methyl ether methacrylate (PEGMA) segments and linear poly (trifluoroethyl methacrylate)‐*co*‐poly (poly (ethylene glycol) methyl ether methacrylate copolymer (LCP).^[^ ^101^ ^]^ Benefiting from the improved compatibility of the modified nano‐SiO_2_ and the LCP copolymer, the ionic conductivity, Li^+^ transference number of the electrolyte, and cycling performance of the assembled cell have been improved (Figure 10a). Overall, these results indicate that nanomaterials are indeed a suitable additive for solid‐state electrolytes to improve performances.

Besides, the quasi‐solid‐state composite star polymer electrolyte can also show excellent performance. Guan et al. reported a high‐performance quasi‐solid‐state star brush block copolymer electrolytes (SBBCEs) composed of two‐arm star polymer of [poly[poly(ethylene glycol) methyl ether acrylate]‐ *b*‐polystyrene]_2_, fumed SiO_2_ and LiTFSI (Figure 10b).^[^ ^102^ ^]^ The obtained SBBCEs without any chemical crosslinker could have a considerable mechanical strength with the compression modulus of 0.35 MPa and high ionic conductivity of 2.1 × 10^−4^ S cm^−1^ at 28 °C because of the synergistic effect between organic–inorganic components, as shown in Figure 10b. And the cells with LiFePO_4_ cathode could deliver a better performance. As for the cell with Ni‐rich cathode LiNi_0.89_Co_0.09_Mg_0.02_O_2_, it was found that its maximum discharge capacities at 28 °C were 159.7 mAh g^−1^ at 0.2 C and 133.0 mAh g^−1^ at 0.5 C, presenting the excellent cycling performance with high reversible capacities.

Particularly, for the preparation method, electrospinning is a low‐energy‐consumption and high‐efficiency method to obtain high porosity and mechanical strength separators. In this regard, Wang et al. found a way to take advantage of hyperbranched star polymers and electrospinning ceramic nanowires (Figure 10c). They utilized hyperbranched polyethylenimine (HPEI) as the core and polyglycolate‐polycaprolactone block copolymer (PGC‐*b*‐PCL) as arms of the polymer electrolyte matrix (HPEI–PGC–PCL).^[^ ^103^ ^]^ On one hand, the HPEI containing N atoms is beneficial to the dissociation of lithium salts. On the other hand, the ceramic nanofillers can provide fast ion transport channels and required mechanical strength. Thus, the composite system could exhibit excellent comprehensive performances. The ionic conductivity of the CSPE from 30 to 80 °C could reach 2.66 × 10^−5^ S cm^−1^ and 5.36 × 10^−4^ S cm^−1^, respectively. It was shown that the discharge capacity of LFP/CSPE/Li coin cell was kept at 162 mA h g^−1^ at the C‐rates of 0.2, 0.5, and 1 C, while had an average discharge capacity of 158 mA h g^−1^ with an average coulombic efficiency of 99.8% over 200 cycles at 0.5 C. As for the high‐voltage cathode LiCoO_2_, the assembled battery also exhibited very good cycling performance and rate capacity under a high active substance content (≈4.5 mg cm^−2^) of the cathode.

**Figure 11:**
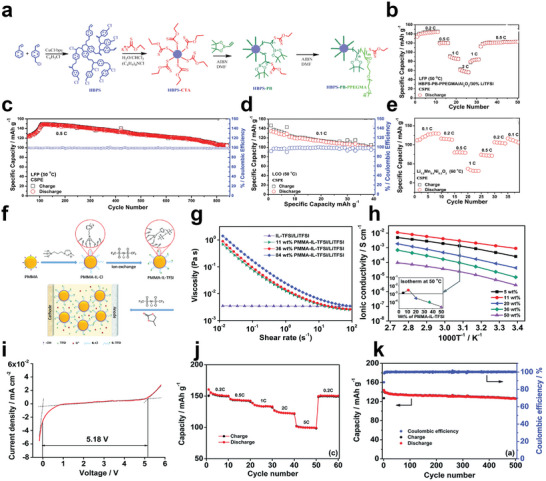
a) Synthetic route of HBPS‐PB‐PPEGMA by sequential RAFT polymerization; b) the corresponding cycle performance of the LFP/CSPE/Li cell at 0.2, 0.5, 1, and 2 C. The test temperature was 50 °C; c) long cycle performance of the LFP/CSPE/Li cell after the high C‐rates cycling; d) cycle performance of the LCO/CSPE/Li cell. The experiments were conducted at 50 °C; e) rate performance of the Li_1.2_Mn_0.5_Ni_0.2_O_2_/CSPE/Li cell. The experiments were conducted at 60 °C. Reproduced with permission.^[^ ^104^ ^]^ Copyright 2018, Elsevier B.V. f) Procedure used to prepare PMMA‐IL‐TFSI/LiTFSI mixtures; g) shear rate dependencies of viscosity for IL‐TFSI/LiTFSI and SIPEs with different contents of PMMA‐IL‐TFSI at room temperature under steady shear; h) ionic conductivities of various weight fractions of PMMA‐ILTFSI in PMMA‐IL‐TFSI/LiTFSI SIPEs as a function of temperature. Inset: isothermal ionic conductivities at 50 °C; i) *I*–*V* diagram obtained from linear‐sweep voltammetry of the 11 wt% PMMA‐IL‐TFSI/LiTFSI SIPE in a Li metal cell. The measurement was performed at a scan rate of 1 mV s^−1^ at room temperature; j) rate capability of Li|11 wt% PMMA‐IL‐TFSI/LiTFSI|LTO cells at 0.2, 0.5, 1, 2, and 5 C; k) cycling performance and coulombic efficiency at 1 C. Reproduced with permission.^[^ ^105^ ^]^ Copyright 2016, The Royal Society of Chemistry.

In another work, Wang et al. combined the Al_2_O_3_ nanowires with the hyperbranched star polymer electrolyte matrix (HBPS‐PB‐PPEGMA) (**Figure** **11**a).^[^ ^104^ ^]^ Owing to the introduction of poly (pinacol vinylboronate) segments into the hyperbranched star polymer matrix, the electrochemical stability of the composite system could be greatly improved. Thus, the star‐shaped composite polymer electrolyte showed an outstanding performance in ionic conductivity, which was 9.63 × 10^−5^ S cm^−1^ and 6.90 × 10^−4^ S cm^−1^ at a given temperature from 30 to 80 °C, respectively. The oxidative decomposition peak could reach 5.2 V, denoting that the CSPE had very good electrochemical stability. And it was found that the cell equipped with the CSPE and LFP cathode had the same charge and discharge capacities at the given C‐rate (Figure 11b). Even after 840 cycles at 0.5 C, the cell still had a discharge capacity of 104 mAh g^−1^ with a coulombic efficiency of 99.56% (Figure 11c). As for the high‐voltage cathodes, it was found that both the LCO‐cell system (Figure 11d) and LMN‐cell system (Figure 11e) could present the excellent cell performance, indicating the outstanding comprehensive performance of the composite polymer electrolyte.

To further improve the practicality of the polymer electrolytes, a novel single‐ion conducting polymer electrolyte (SIPE) (Figure 11f) based on ionic liquid (IL)‐decorated PMMA nanoparticles dispersed in propylene carbonate (PC) host was developed by Li et al.^[^ ^105^ ^]^ The obtained SIPE with shear‐thinning behavior (Figure 11g) achieved a remarkable ionic conductivity (Figure 11h) of 3.13 × 10^−3^ S cm^−1^ at room temperature with a wide electrochemical window up to 5.18 V (Figure 11i). Furthermore, the assembled Li/Li_4_Ti_5_O_12_ half cells exhibited excellent rate performance (Figure 11j). Even increasing the current density to 5 C, the discharge capacity remained at 100 mA h g^−1^. In terms of long cycling performance, the battery presented a highly reversible capacity of about 126 mA h g^−1^ at 1 C with a coulombic efficiency of 99% after 500 cycles (Figure 11k). Importantly, the operating life of the battery has increased by almost ten times with the addition of as little as 11 wt% PMMA‐IL‐TFSI, which means the introduction of unique structures indeed plays a role in improving electrolyte performances.

Overall, nanomaterials are the promising materials to add to the polymer electrolyte matrix in order to further inhibit the crystallinity of polymer segments and enhance the poor mechanical properties. Noteworthily, the amounts of nanoparticles in the polymer matrix needs to be controlled, because the particle agglomeration and phase separation caused by the nanofillers in high concentration will further reduce the efficiency of ion transportation as well as the mechanical strength of the composite polymer electrolyte. As an effective solution, ceramic nanowires obtained by electrospinning can be the substitutional fillers in composite system due to its high length‐diameter ratio and the ability to construct fast ion channels.

In summary, functional star polymer matrix, plasticizers, and nanomaterials are all common additives that can be used in composite star‐shaped polymer electrolyte system. It should be noted that each additive has its advantages and its limitations. To obtain the optimal performance of the electrolyte membrane, it is necessary to fully consider the interaction between the additive and the polymer electrolyte matrix.

## 4 Comb‐Like Type Polymer Electrolytes

To improve the ionic conductivity and mechanical properties of linear polymer electrolytes, the grafting of functionalized side chains on the linear main chain can inhibit the crystallization of the polymer and increase the mobility of the segments. Comb‐like type polymers as one of the topological polymers, which are formed by grafting multiple linear branches onto the main chain can also be used as electrolyte matrix.

### 4.1 Comb‐Like Type Polymer Electrolytes Based on Different Polymer

Depending on the diverse polymer backbones, the reported comb‐like type polymer electrolytes are common in the following categories.

#### 4.1.1 Benzene‐Containing Polymer

**Figure 12:**
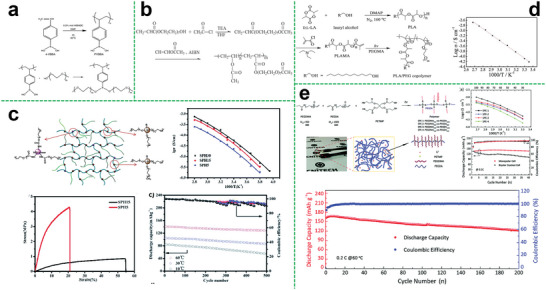
a) Synthesis illustration of PVBBA and scheme of comb‐branched copolymers. Reproduced with permission.^[^ ^106^ ^]^ Copyright 2010, Elsevier B.V. b) Synthetic route of the AEOA monomer and copolymer. Reproduced with permission.^[^ ^108^ ^]^ Copyright 2010, John Wiley & Sons, Ltd. c) Structure of the Si‐PEG‐HDI trimer (SPH) comb polymer network; temperature dependence of ionic conductivity of SPH30, SPH15, and SPH5; stress–strain curves for SPH15 and SPH5; the capacity retentions and coulombic efficiency of the LiFePO_4_/SPH15/Li cells with a 0.5 C current rate at 60, 30, and 10 °C. Reproduced with permission.^[^ ^110^ ^]^ Copyright 2017, The Royal Society of Chemistry. d) Schematic representation for the preparation of comb‐like PLA/PEG copolymer; Arrhenius plots for PLA/PEG‐SPE at the [EO/Li] ratio of 32:1. Reproduced with permission.^[^ ^111^ ^]^ Copyright 2020, The Royal Society of Chemistry. e) Route used for the synthesis of the copolymer via “thiolene” click chemistry; Arrhenius plots for SPEs with EO/Li^+^ =20:1; cycling performance of monopolar and bipolar ASSLBs at 0.5 C at 60 °C; cycling performance at 0.2 C at 60 °C and 0.1 C at ambient temperature. Reproduced with permission.^[^ ^112^ ^]^ Copyright 2018, The Royal Society of Chemistry.

The benzene in the polymer backbones could provide the mechanical strength of polymer electrolyte, the prepared electrolyte membrane is beneficial to possess the characteristics of self‐supporting. Especially, the flexible side chains facilitate to promote the electrochemical property of the electrolyte. Çelik et al. reported a comb‐branched copolymer (**Figure** **12**a) that utilized poly(4‐vinylbenzeneboronic acid) as the boron‐containing main chain and followed by modification with polyethylene glycol monomethylether (PEGME) as the ion‐conducting segments.^[^ ^106^ ^]^ It was found that the ionic conductivity of these polymer electrolytes depended on the side chain length as well as the Li^+^ content. And the satisfactory value of ionic conductivity is 1.6 × 10^−4^ S cm^−1^ at 20 °C.

#### 4.1.2 Polyester

The backbones with polyester would perform great at flexibility and ion conduction. It was reported that comb‐like type polymer electrolyte based on pendant polar groups could exhibit higher ionic conductivity at room temperature.^[^ ^107^ ^]^ Zhou et al. prepared the comb copolymer electrolyte (Figure 12b) with pendant polar carbonyl groups via the copolymerization of acetyl‐oligo (ethylene oxide) acrylate (AEOA) and vinyl acetate (VAc).^[^ ^108^ ^]^ The introduction of carbonyl groups into the flexible polymer matrix could accelerate the dissociation of lithium salts, resulting in the enhancement of ionic conductivity. As a result, the ionic conductivity of the comb‐like type polymer electrolyte could reach 1.2 × 10^−4^ S cm^−1^ at room temperature with the VAc concentration of 14.3 wt% and LiClO_4_ at 12 wt%.

#### 4.1.3 Polyurethane

Unlike the flexible polymer segments used in the above work, thermoplastic polyurethane is also the desirable polymer matrix for electrolytes. Thermoplastic polyurethane has two‐phase microstructure: the soft segments as ion‐conducting units can dissolve alkali metal salt, resulting in good ionic conductivity, while the hard segments act as the rigid scaffold to achieve mechanical strength. In this regard, Bao et al. fabricated the comb‐like nonionic waterborne polyurethane (NWPU) via a solvent‐free method using isophorone diisocyanate, poly(propylene oxide glycol) (PPG) and poly(ethylene glycol monomethyl ether)‐based trimethylolpropane (MPEG‐diol).^[^ ^109^ ^]^ The NWPU‐based polymer electrolyte showed sufficient mechanical strength and good electrochemical performance. The maximum stress of the SPE was 5.63 MPa and its elongation at break could achieve 640%. The ionic conductivity of the SPE with the mass content of 15% LiClO_4_ could reach 5.44 × 10^−6^ S cm^−1^ at 40 °C and 2.35 × 10^−3^ S cm^−1^ at 140 °C, respectively.

Thus, the work illuminates that polyurethane may be a suitable polymer host for preparing the SPEs with enhanced ionic conductivity and sufficient mechanical strength.

#### 4.1.4 Si‐Doped PEG

Compared with the traditional carbon‐based polymer matrix, the Si‐doped copolymers as electrolyte matrix can show better flexibility due to its lower glass transition temperature. In this regard, a comb‐like Si‐PEG copolymer electrolyte (Figure 12c) with a cross‐linked structure was synthesized.^[^ ^110^ ^]^ The comb‐like polymer electrolyte exhibited an ionic conductivity of 1.2 × 10^−4^ S cm^−1^ at 30 °C and 3.2 × 10^−5^ S cm^−1^ at 10 °C, and the electrolyte has a higher Li^+^ transference number up to 0.62 due to the promoted ion mobility, as shown in Figure 12c. Besides, the tensile strength of 0.8 MPa at 30 °C could be also obtained. Furthermore, the Si‐PEG polymer electrolyte also showed good long‐cycling and rate performance in a wide temperature range, from 10 to 60 °C. The assembled LFP/Li battery could deliver the specific capacities of 84 mAh g^−1^ at 10 °C and maintain 75% capacity after 500 cycles at 0.5 C, indicating the beneficial effects of functional heteroatoms in improving the performance of the electrolytes.

#### 4.1.5 Polylactide

Owing to its good modulus, polylactide is also a potential host for comb‐type polymer electrolytes. Zaheer et al. reported an in situ polymerized comb‐like copolymer based on methyl acrylate functionalized polylactide (PLAMA) and poly(ethylene glycol) methyl ether methacrylate (PEGMA).^[^ ^111^ ^]^ The polymer electrolyte had a remarkably high ionic conductivity value of 6.9 × 10^−5^ S cm^−1^ at ambient temperature and a maximum ionic conductivity of 4.3 × 10^−4^ S cm^−1^ at 60 °C (Figure 12d).

#### 4.1.6 Polyether by Thiol‐Ene/Alkene Reaction

This type of polymer electrolyte can adopt a simpler and more environmentally friendly preparation method. In general, the traditional solution casting method with the organic solvent as the media usually cause uneven film‐forming characteristics, which has an impact on the performance of electrolyte membranes. In comparison, the solvent‐free UV‐cured method is more environmentally‐friendly and high‐efficient. Due to the features of low temperatures, solvent‐free processes, and fast conversion, the solvent‐free UV photopolymerization is chosen to prepare the high‐performance electrolytes. In this regard, a series of comb‐like polymer electrolytes based on poly (ethylene glycol) methyl ether methacrylate (PEGDMA), poly (ethylene glycol) diacrylate (PEGDA), and pentaerythritol tetra(3‐mercaptopropionate) (PETMP) were prepared via “thiol‐ene” click chemistry (Figure 12e).^[^ ^112^ ^]^ The highest ionic conductivity of the comb‐like polymer electrolytes was 5.05 × 10^−5^ S cm^−1^ at ambient temperature, while a higher Li^+^ transference number could reach 0.312 at 60 °C. Besides, the LiFePO_4_‐based battery delivered a maximum capacity of 167.39 mA h g^−1^ and remained at 122.17 mA h g^−1^ after 200 cycles at 60 °C. And it could exhibit good discharge capacities at room temperature. Also, the assembled bipolar cell could present excellent safety and cell performance, as shown in Figure 12e.

**Figure 13:**
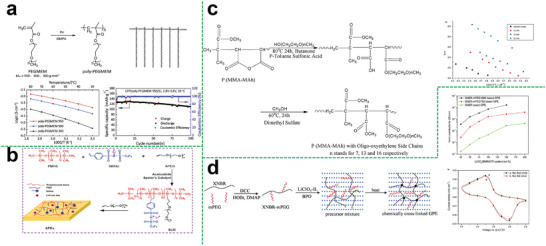
a) Chemical synthetic route of comb‐like polymers; Arrhenius plots for different SPEs with EO/Li^+^=18:1; the cycling performance of LFP/poly‐PEGMEM 950/Li soft package cells at 0.2 C under 30 °C. Reproduced with permission.^[^ ^113^ ^]^ Copyright 2019, The Chinese Ceramic Society. b) Preparation process of solid‐state single‐ion conducting polymer electrolytes (SPEs). Reproduced with permission.^[^ ^114^ ^]^ Copyright 2017, Wiley Periodicals, Inc. c) Synthesis of comb‐like MMA/MAh‐*g*‐PEGME copolymers; corresponding VTF plots for the LiClO_4_‐containing polymer electrolytes based on P(MMA‐MAh) and three comb‐like copolymers. Reproduced with permission.^[^ ^115^ ^]^ Copyright 2009, Elsevier B.V. d) Preparation route and schematic structure of GPE; ionic conductivity of GPE based on XNBR, XNBR‐mPEG750, and XNBR‐mPEG1900 at 30 °C; the second and third circle of cyclic voltammogram with LiFePO_4_/GPE/Li cell using XNBR‐mPEG1900/150% LiClO_4_‐IL as electrolytes. Reproduced with permission.^[^ ^116^ ^]^ Copyright 2012, Springer Science Business Media B.V.

Likewise, a novel polymer electrolyte with the comb‐like structure based on poly (ethylene glycol) methyl ether methacrylate (PEGMEM) and Lithium bis (trifluoromethane) sulfonilimide (LTFSI) was prepared via the solvent‐free UV‐cured method (**Figure** **13**a).^[^ ^113^ ^]^ Because of the uniform distribution of the components during solvent‐free film formation, such electrolytes presented a considerably high conductivity of 1.44 × 10^−4^ S cm^−1^ and a wide electrochemical window of 5.4 V at 30 °C, as shown in Figure 13a. Besides, the LiFePO_4_‐based battery exhibited good rate performance. When the temperature dropped to 30 °C, the battery could also present good discharge specific capacities at different C‐rates. Moreover, the soft package battery also showed a good cycling performance (Figure 13a).

#### 4.1.7 Polysiloxane

However, the electrolytes mentioned above are all dual‐ion conductors, in which lithium ion and anion of lithium salt can move simultaneously, easily leading to concentration gradients and causing battery failure. In this regard, the advent of single ion polymer electrolytes is expected to solve this problem. A novel comb‐like siloxane copolymer (Figure 13b) based on pendant lithium 4‐styrenesulfonyl (perfluorobutylsulfonyl) imide and poly (ethylene glycol) was reported.^[^ ^114^ ^]^ Owing to the enhanced segments mobility of more flexible Si‐O units as well as the weak association between ‐SO_2_‐N^(−)^‐C_4_F_9_ and Li‐ions, the designed polymer electrolyte possessed promising properties such as ultra‐low glass transition temperature, high conductivity of 3.7 × 10^−5^ S cm^−1^ and the improved Li‐ion transference number of 0.80, which is much higher than that of dual‐ion conductors.

Taken together, it can be found that a free‐standing electrolyte membrane with both good electrochemical and mechanical properties can be successfully obtained by adjusting the ratio between the ion conductive segments and the rigid units in the comb polymer electrolyte systems. And the different side chains and synthetic approaches influence the properties in different ways. However, some pristine comb‐like type polymer electrolytes are still far from satisfactory in terms of excellent battery performances. In this regard, introducing other functional additives into the abovementioned systems is one of the effective methods to further improve the comprehensive performances of the electrolyte membranes.

### 4.2 Composite Comb‐Like Type Polymer Electrolytes

Common additives used in composite comb‐like type polymer electrolytes are inorganic liquid additives such as liquid electrolytes or ionic liquid and functional polymer matrix like PVDF or PEO, which can further improve the overall performance of electrolyte membranes.

#### 4.2.1 Polymer‐Liquid Electrolyte

Gel polymer electrolytes (GPEs) possess both the high conductivity of liquid electrolytes and good mechanical properties of solid electrolytes, which have received widespread attention. Wang et al. prepared the methyl methacrylate‐based comb‐like GPE that composes of poly(ethylene glycol) monomethyl ether (PEGME) and liquid electrolyte (PC) with LiClO_4_ dissolved, as shown in Figure 13c.^[^ ^115^ ^]^ Due to the excellent compatibility between the prepared electrolyte and electrodes as well as the wettability of liquid electrolytes, the highest conductivity obtained in such a GPE system was 1.22 × 10^−3^ S cm^−1^ at 60 °C.

#### 4.2.2 Polymer‐Ionic Liquids

Similar to liquid electrolyte, the addition of ionic liquids can also achieve high conductivity. Li et al. designed a comb‐like GPE based on carboxylated butadiene‐acrylonitrile rubber (XNBR), poly(ethylene glycol) monomethylether (mPEG), and ionic liquids (1‐butyl‐3‐methylimidazolium trifluoromethane‐sulfonate) (Figure 13d).^[^ ^116^ ^]^ Compared with traditional polymer matrix, butadiene‐acrylonitrile rubber (NBR) with low glass transition temperature (*T*_g_), non‐crystallinity, and good elasticity can facilitate tight contact of the interface. Thus, the ionic conductivity of the GPE could achieve a maximum value of 1.64 × 10^−3^ S cm^−1^ at 30 °C, as shown in Figure 13d. Besides, the LiFePO_4_‐based cell can also remain working stability in the cyclic voltammogram experiment.

#### 4.2.3 Polymer‐Polymer

Some functional polymers can also be used for the development of composite systems. PVDF and its derivatives show the features of semi‐crystalline and can achieve better dissolution of lithium salts to promote the ionic concentration. Li et al. designed a comb‐like grafted copolymer electrolyte P(MMA‐*co*‐PEGMA), the copolymer P(MMA‐*co*‐PEGMA) was synthesized by combining methyl methacrylate (MMA) with poly(ethylene glycol) methacrylate (PEGMA) via radical polymerization.^[^ ^117^ ^]^ Then, the polymer electrolyte was obtained by mixing the P(MMA‐*co*‐PEGMA) and liquid electrolyte with PVDF matrix. Particularly, the maximum conductivity of the GPE could reach 3.01 × 10^−3^ S cm^−1^ at 25 °C. Also, the composite electrolyte system had the highest stress of 3.24 MPa. Besides, the Graphite|GPE|LiFePO_4_ cell exhibited improved capacity retention at various C‐rates and higher coulombic efficiency than pure PVDF separators.

**Figure 14:**
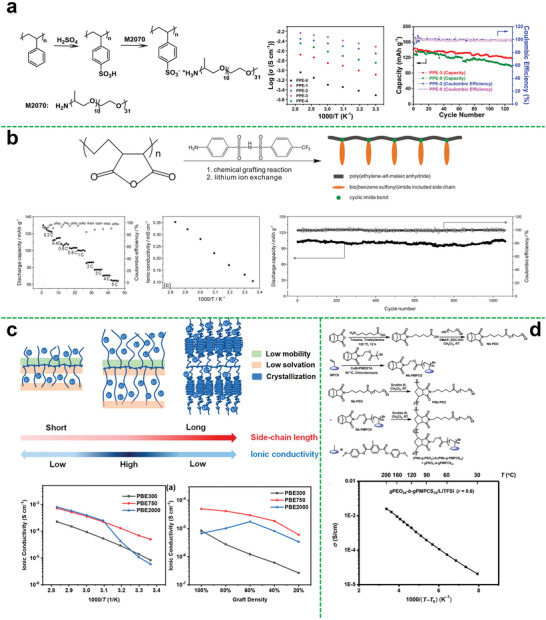
a) Synthesis route of the grafted copolymer modified by ionic bond and the chemical structure of M2070; temperature dependence of ionic conductivity for PPEs with various mass fractions of grafted polymers; specific discharge of PPE‐3 based LiFePO_4_/Li battery at 0.1 C under room temperature. Reproduced with permission.^[^ ^118^ ^]^ Copyright 2018, Elsevier B.V. b) Side grafting functional groups on a selected commercial polymer; rate capability of the Li/electrolyte/LiFePO_4_‐C type coin cell; ionic conductivity of the blend polymer electrolyte film; cycle performance at 1 C under room temperature. Reproduced with permission.^[^ ^119^ ^]^ Copyright 2016, Elsevier. c) Schematic illustration of the heterogeneous distribution of solvation probability and segmental mobility, and the varied side chain topology of the PBEs with increasing side chain length; plots of ionic conductivity of PBE300, PBE750, and PBE2000 vs inverse temperature; plot of ionic conductivities of PBE300, PBE750, and PBE2000 vs graft densities at 25 °C. Reproduced with permission.^[^ ^119^ ^]^ Copyright 2022, American Chemical Society. d) Synthesis of BBCPs; the plot of the logarithmic scale of *σ* of the complex vs 1/(T‐T_0_). Reproduced with permission.^[^ ^120^ ^]^ Copyright 2017, American Chemical Society.

Besides, a porous comb‐like polymer electrolyte (**Figure** **14**a) with ionic bonds was prepared successfully.^[^ ^118^ ^]^ The electrolyte with ionic bonds could enhance the molecular motion than that with the covalent bond. Thus, the ionic conductivity of the obtained electrolyte was increased to 3.05 × 10^−3^ S cm^−1^ at 30 °C. The electrolyte also had a wide electrochemical stability window up to 4.8 V. Meanwhile, the LFP‐based cell possessed an initial discharge capacity of 141 mA h g^−1^ and 96% capacity retention after 130 cycles, and the cell also presented good rate performance.

However, for lithium‐ion batteries, lithium dendrite formation owing to the concentration polarization has been still recognized as the obstacle limiting the further improvement of dual‐ion conductor electrolyte systems. In this regard, the single ion‐conductors can solve the above problems as a feasible improvement strategy for the enhancement of performance. Pan et al. reported a comb‐like single‐ion polymer electrolyte (Figure 14b) by blending lithiated ionomers with poly(vinylidene fluoride‐*co*‐hexafluoropropylene) (PVDF‐HFP).^[^ ^119^ ^]^ Especially, the flexible alkyl main chain of ionomers provides mechanical stability, while the functional polar side chains are beneficial for lithium ionization. Thus, the obtained polymer electrolyte had an ionic conductivity of 0.104 mS cm^−1^ at room temperature, as shown in Figure 14b. Besides, the synthesized electrolyte had a good mechanical performance, the tensile strength can reach up to 15.5 MPa and percent elongation at break of 5%. And the lithium‐ion transference number of the composite polymer electrolyte could reach 0.92, indicating the inhibition of concentration polarization. Besides, the assembled cells could deliver a reversible discharge capacity of 100 mAh g^−1^ at 1 C for 1000 cycles without obvious decay, displaying an excellent rate and long‐term cycle performance.

Besides, the inorganic fillers are also applied in the polymer electrolyte, Feng et al. added halloysite nanotube (HNT) to comb‐like methoxy poly(ethylene glycol) acrylate polymer electrolyte (HCPE) for making a composite electrolyte.^[^ ^50^ ^]^ Similar to most inorganic fillers, the addition of HNT greatly enhances the ion transport capacity of electrolyte and mechanical strength.

Overall, comb‐like type polymers are also an appealing host for the preparation of polymer electrolytes with excellent overall comprehensive performances. On one hand, the branched structure can inhibit the crystallization of the polymer matrix and the existence of more functional groups further promotes the dissociation of lithium salt. On the other hand, the cooperation of the flexible segments and the rigid segments of comb‐like polymers can also maintain a certain mechanical strength. Thus, the comb‐like type polymer electrolytes are expected to have both the outstanding electrochemical and mechanical properties.

## 5 Brush‐Like Type Polymer Electrolytes

Brush‐like type polymers or polymer brushes refer to a special polymer structure formed by grafting polymer molecular chains with higher density and a certain length on the surface of the substrate or the polymer main chain. Polymer brushes have attracted widespread attention in the field of solid‐state polymer electrolytes due to their unique topologies and functions.

### 5.1 Brush‐Like Type Polymer Electrolytes Based on Different Polymer

#### 5.1.1 Polyether

The side chain architectures of brush‐like type polymer have strong effect on the entire structure, both the chain length and graft density can influence mechanism. When the polymer is used in lithium ion batteries as electrolyte, it can further impact battery properties. Ji et al. prepared a series of polymer‐brush electrolytes (PBEs) with various PEO side chain lengths and graft densities to study the relationship between the side chain architectures and the ion‐conducting behaviors of PBEs. From results, the ionic conductivity was nonmonotonic and varied with different PEO side chain length, and the amorphous PBEs achieve the highest ionic conductivity compared with other control groups. The PBEs with amorphous side chains (≤750 Da) achieved the highest ionic conductivity at 100% graft density (Figure 14c).

#### 5.1.2 Polyester/Polycarbonate

Ping et al. developed a series of brush block copolymers (BBCPs) via tandem ring‐opening metathesis polymerization (ROMP).^[^ ^120^ ^]^ As shown in Figure 14d, it was found that the most ordered lamellar structure would be formed if the rigid units and PEO soft side chains were the same lengths, leading to higher ionic conductivity at ambient temperature. Thus, the value of ionic conductivity could reach 1.58 × 10^−3^ S cm^−1^, which was one of the highest values for PEO‐based polymer electrolytes.

Normally, traditional polymer electrolytes usually suffer from unpredictable breakage or crack under the influence of external force during normal cycling of battery, resulting in severe safety issues. In this regard, polymers with the ability of self‐healing have attracted widespread attention. It was reported that self‐healing refers to the ability to recover mechanical damage through different interactions including hydrogen bonding, host‐guest interaction, or reversible chemical bonds.^[^ ^121^, ^122^, ^123^, ^124^, ^125^ ^]^

**Figure 15:**
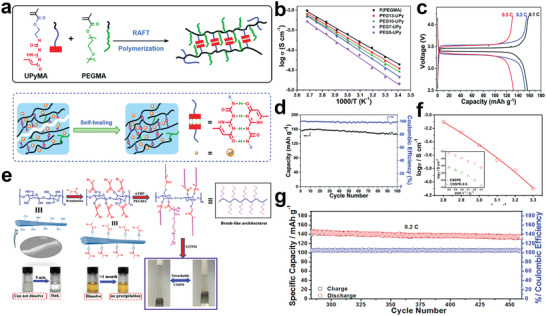
a) Schematic illustration on the synthesis of brush‐like copolymer and the formation of self‐healing PE and the self‐healing mechanism between cut surface; b) the ionic conductivities of PEs based on copolymers and the P(PEGMA); the solid lines represent Arrhenius fitting results; c) initial charge and discharge profiles of the batteries at different rates under 60 °C; d) cycling performance of LFP/shPE/Li cell at 0.1 C. Reproduced with permission.^[^ ^126^ ^]^ Copyright 2018, The Royal Society of Chemistry. e) Synthetic route of cellulose‐based polymer with brush‐like architectures. The figure at the bottom right shows that the prepared CSSPE exhibits excellent flexibility and stretchability; f) ionic conductivity of CSSPE at a given temperature, which is compared with CSSPE‐0.5; g) cycle performance of the LFP/CSSPE/Li cell at 0.2 C. Reproduced with permission.^[^ ^127^ ^]^ Copyright 2020, American Chemical Society.

Zhou et al. designed a self‐healing and highly stretchable polymer electrolyte (shPE) with a physically cross‐linked network via ureidopyrimidinone (UPy) contained brush‐like poly(ethylene glycol) chains (**Figure** **15**a).^[^ ^126^ ^]^ Due to the interaction of intermolecular hydrogen bonds, the electrolyte membrane could heal the cut damage within 2 h under the ambient condition without any external stimulus and be stretched to more than 20 times longer without break. Also, the cell presented a good ionic conductivity and good rate and cycling performance at 60 °C (Figure 15b–d).

#### 5.1.3 Cellulose

Cellulose is a natural polymer material with excellent mechanical strength, which can be used as the molecular skeleton and potential ion channels owing to its tendency to form ordered microstructures along with specific directions.

Inspired by this, a highly stretchable brush‐like cellulose‐based polymer electrolyte with brush‐like architectures (Figure 15e) was prepared by Wang et al.^[^ ^127^ ^]^ The cellulose‐based polymer electrolyte presented an excellent mechanical property (stretchability >150%) and a good ionic conductivity of 8.00 × 10^−5^ S cm^−1^ at 30 °C (Figure 15f). Moreover, the discharge capacity of the LFP/cellulose‐based SPE/Li type cell could maintain at 140 mAh g^−1^ with the coulombic efficiency of over 99% after 450 cycles at 0.2 C (Figure 15g).

In conclusion, brush‐like type polymer electrolytes are very promising for solid‐state lithium‐ion batteries. By using the molecular microstructure and intermolecular interactions of different building blocks, the overall performance of the electrolyte membranes can be greatly improved, while exhibiting some attractive features like the ability of self‐healing. Without the reduction of electrochemical performance, self‐healing is surely an appealing feature for realizing the practicability and security of polymer electrolytes.

### 5.2 Composite Brush‐Like Type Polymer Electrolytes

**Figure 16:**
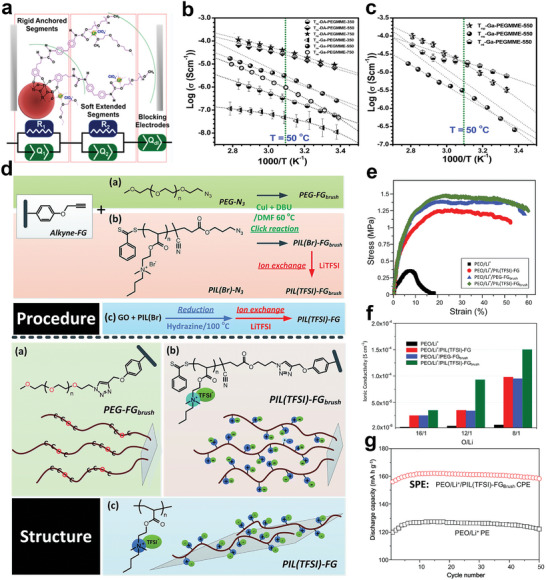
a) Schematic diagram of the polymer‐nanocomposites with brush‐like architectures; typical log(*σ*) vs 1000/T Arrhenius plots depicting the dependence of ionic conductivity as a function of temperature showing: b) the effect of different functional groups and chain length of the brush with titania nanospindles as the core, c) the effect of different morphology of the titania nanostructures used. LiClO_4_ at EO/Li=20 is solvated in the composite electrolyte matrices. Reproduced with permission.^[^ ^128^ ^]^ Copyright 2014, American Chemical Society. d) Schematic diagram for the synthesis of polymer‐functionalized RGO brush (polymer‐FG_brush_) and PIL(TFSI)‐FG; e) stress–strain curves of PEO/Li^+^ PE and PEO/Li^+^/polymer‐FG 0.6 wt% CPEs; f) ion conductivity of PEO/Li^+^ PEs at 60 °C with 0.6 wt% polymer‐FG contents at various salt concentrations; g) cyclic performance of Li/SPE/LiFePO_4_ cell at 60 °C. Reproduced with permission.^[^ ^129^ ^]^ Copyright 2015, Royal Society of Chemistry.

Nanomaterials are also used in the preparation of composite electrolytes by combing with brush‐like type polymers because of their special characteristics. Gowneni et al. reported the polymer‐nanocomposites with brush‐like architectures (**Figure** **16**a) based on the nanostructured titania and polyethylene glycol.^[^ ^128^ ^]^ They studied several issues in this field including core morphology, surface modifiers/functionality, grafting length, and density of the brushes (Figure 16b,c). The study contributes to our understanding of the abovementioned unique architecture, which could facilitate maintaining the relatively low glass transition temperature and excellent mechanical stability while increasing the amorphous nature of the ion‐conduction pathways within a solid matrix. Also, this pioneering work revealed the potential applications of nanostructured hybrid electrolytes and provided new insight into the design of polymer electrolyte molecular structures.

Graphene oxide (GO), used as another type of nano‐fillers in the composite system, has also been seen as potential candidate materials for enhancing the ionic conductivities and mechanical properties. In this regard, a reduced GO‐based 2D molecular brushes with PIL arms [PIL(TFSI)‐FG_brush_] (Figure 16d) was reported and then blended with PEO matrix to prepare the composite polymer electrolytes.^[^ ^129^ ^]^ The role of [PIL(TFSI)‐FG_brush_] is to optimize the ionic transfer conditions in the PEO matrix. The synthesized electrolyte delivered better ion conductivity and battery performance (Figure 16f,g). Thus, the CPE presented a tensile strength of 1.45 ± 0.08 MPa, leading to more than 300% improvement in the tensile strength of the pure one (Figure 16e).

The introduction of nano‐fillers is beneficial for the improvement of electrochemical and mechanical properties in the system of composite brush‐like type polymer electrolytes. Nanomaterials with the modification of organic components can further improve the compatibility with the polymer matrix and provide a fast channel for effective ion conduction. Thus, composite brush‐like type polymer electrolytes had high ionic conductivities and excellent mechanical properties, which are promising for lithium‐ion batteries.

## 6 Summary and Prospects

The advent of SPEs is expected to address the potential safety issues of liquid electrolytes. Up to date, tremendous efforts have been put into these fields to further simultaneously improve both the ionic conductivity and mechanical strength of the existing SPEs and interfacial compatibility.^[^ ^130^, ^131^ ^]^ As a method to solve the above problems in essence, the design of the molecular structure of the polymer electrolytes matrix has attracted more and more attention. From this perspective, nonlinear topological polymers like hyperbranched, star‐shaped, comb‐like, and brush‐like type polymers have become the most promising materials for solid‐state polymer electrolytes due to their unique topological structure, amorphous, high solubility in common organic solvents, easy film‐formation, and containing a large number of functional groups that can be further modified. Therefore, employing various strategies such as structural design of the polymer matrix or blending, the room temperature ionic conductivity, mechanical strength, and even the overall performance of the topological polymer electrolytes is hopeful to be greatly improved. In this review, the current progress and development of the topological polymer electrolytes during the past decades have been highlighted. Although many advances in this field have been achieved, there are still a few challenges that have to be faced. Specifically, the development of electrolytes that possess high lithium storage behavior and higher working voltage has been seen as the priority accompanied by the increasing demands for higher energy density storage devices. For now, obtaining the solid‐state electrolyte with excellent performance for practical commercial application is still a challenge. Thus, it is important to develop novel electrolytes that keep pace with the times.

In this regard, future research on topological polymer electrolytes will mainly focus on the following aspects: 1) design of new structure to achieve kinetic and thermodynamic stability at the electrolyte‐electrode interfaces; 2) exploration of the mechanism of ion transport by the combination of experiment and theoretical calculation; 3) development of more attractive additives to integrate the performance of existing electrolytes; 4) preparation of new lithium salt to improve electrochemical performance; 5) exploration of new techniques like optimizing the battery design structure to improve the overall performances; 6) reforming the present preparation methods or improving the film‐forming processes to realize the applications of topological polymer electrolytes; 7) further development of new functional electrolyte including self‐healing and highly stretchable polymer electrolyte to be suitable for flexible wearable devices. Moreover, all aspects above can be most practical for further applications of topological polymer electrolytes.

In summary, the outstanding performances of topological polymer electrolytes make it possible to be used in the next‐generation high energy density lithium‐ion batteries. Even to this day, more and more researchers do their utmost to boost their progress. Theoretical predictions and operational improvements in practice shed new light on the development of SPEs. Although there is still a tough and long way to explore, the studies on the development of high‐performance electrolytes will never be suspended. As we expected, all‐solid‐state lithium‐ion batteries with various excellent performances will be developed and applied in our daily life.

## Conflict of Interest

The authors declare no conflict of interest.
